# Microevolutionary mechanism of high‐altitude adaptation in Tibetan chicken populations from an elevation gradient

**DOI:** 10.1111/eva.13503

**Published:** 2022-10-31

**Authors:** Hai‐An Zhong, Xiao‐Yan Kong, Ya‐Wen Zhang, Yan‐Kai Su, Bo Zhang, Li Zhu, Hua Chen, Xiao Gou, Hao Zhang

**Affiliations:** ^1^ National Engineering Laboratory for Animal Breeding, Beijing Key Laboratory for Animal Genetic Improvement, College of Animal Science and Technology China Agricultural University Beijing China; ^2^ School of Life Science and Engineering Foshan University Guangdong China; ^3^ College of Animal Science and Technology Yunnan Agricultural University Kunming China; ^4^ Center for Computational Genomics Beijing Institute of Genomics, Chinese Academy of Sciences Beijing China

**Keywords:** demographic history, high‐altitude adaptation, microevolution, Tibetan chicken

## Abstract

As an indigenous breed, the Tibetan chicken is found in highland regions and shows physiological adaptations to high altitude; however, the genetic changes that determine these adaptations remain elusive. We assumed that the microevolution of the Tibetan chicken occurred from lowland to highland regions with a continuous elevation range. In this study, we analyzed the genome of 188 chickens from lowland areas to the high‐altitude regions of the Tibetan plateau with four altitudinal levels. Phylogenetic analysis revealed that Tibetan chickens are significantly different from other altitude chicken populations. Reconstruction of the demographic history showed that the migration and admixture events of the Tibetan chicken occurred at different times. The genome of the Tibetan chicken was also used to analyze positive selection pressure that is associated with high‐altitude adaptation, revealing the well‐known candidate gene that participates in oxygen binding (*HBAD*), as well as other novel potential genes (e.g., *HRG* and *ANK2*) that are related to blood coagulation and cardiovascular efficiency. Our study provides novel insights regarding the evolutionary history and microevolution mechanisms of the high‐altitude adaptation in the Tibetan chicken.

## 1 INTRODUCTION

The Qinghai‐Tibetan Plateau (QTP) is well‐known for its high‐altitude and unique environmental characteristics, that is, hypobaric, hypoxic, and strong ultraviolet exposure conditions, which may induce systematic high‐altitude responses in animals living in these areas. The genetic mechanism underlying high‐altitude adaptation is currently a topic of great interest and has important implications for understanding adaptation in low‐oxygen environments. Several animals have been studied to determine the genetic basis of such adaptation using high‐throughput sequencing. For example, whole‐genome selective scans have identified positive selection of a set of genes, including *EPAS1* which is associated with a hypoxia response in Tibetan horses (Liu, Xu, et al., 2019) and Tibetan Mastiffs (Li et al., 2014). Also, selected genes that are linked with metabolic processes (e.g., nutrition metabolism, and energy metabolism) and oxygen transportation have been identified in animals such as yak (Qiu et al., 2012), Tibetan wild boar (Li et al., 2013), and Tibetan sheep (Hu et al., 2019) to explain their high‐altitude adaptation. As an indigenous breed, the Tibetan chicken (TBC) has inhabited the QTP for at least a 1000 years and has adapted well to the hypoxic environment in terms of better embryo survival and higher egg hatchability compared with that of their lowland counterparts (Wu & Li, 2012; Zhang et al., 2008). Besides, the TBC has high‐altitude physiological characteristics such as larger heart and lungs, low right ventricular index, high red blood cell count, hemoglobin concentration, and mean corpuscular volume, which can facilitate oxygen transport and the affinity to overcome hypoxia environment (Wu & Li, 2012; Zhang et al., 2007).

A previous study based on mitochondrial DNA sequence analysis showed that the TBC was not distinguishable from other indigenous chickens in surrounding areas, indicating that the TBC may have diverged from indigenous chickens in lowland areas adjacent to the QTP (Zhang et al., 2017). Recent studies suggested that domestic chickens initially originated from the red jungle fowl (RJF) subspecies *Gallus gallus spadiceus*, which is currently predominant in southwestern China, northern Thailand, and Myanmar (Wang et al., 2020, 2021). However, the migration and adaptative evolution of the TBC remain unclear. The Ancient Tea Horse Road, which formally originated in the Tang Dynasty (618–907), crosses Tibet and Yunnan Province in southwestern China with continuous altitudinal gradients over 3000 m (Sigley, 2010; Yang, 2004). There are several domestic chicken populations living along this route, which might provide a wide spectrum of resources for investigating the microevolution of the TBC.

Studies on the TBC's genetic adaptation to high altitude have primarily focused on comparing highland and lowland populations, and this has resulted in the identification of certain genes that are involved in the Ca^2+^ signaling pathway (Wang, Cheng, & Schmid, 2015; Wang, Li, et al., 2015) and blood vessel development (Zhang et al., 2016). Their results are inconsistent and further study is necessary to reveal the mechanism of high‐altitude adaptation in the TBC using more groups and individuals. In addition, high altitude is not a single point above sea level but, instead, represents a continuum. It has been reported that focusing on a broad elevational continuum is a suitable model system that improves our understanding of the genetic processes that result in high‐altitude adaptation (Gou et al., 2014; Sun et al., 2018). Here, 188 Chinese native chickens that inhabited four continuous altitude levels surrounding the Ancient Tea Horse Road were collected and their whole genomes were sequenced. The phylogenomic structure and gene selections were assessed based on a comparison of highland and lowland pairs, as well as a continuous altitude gradient model.

## 2 MATERIALS AND METHODS

### 2.1 Sample collection and DNA extraction

**FIGURE 1:**
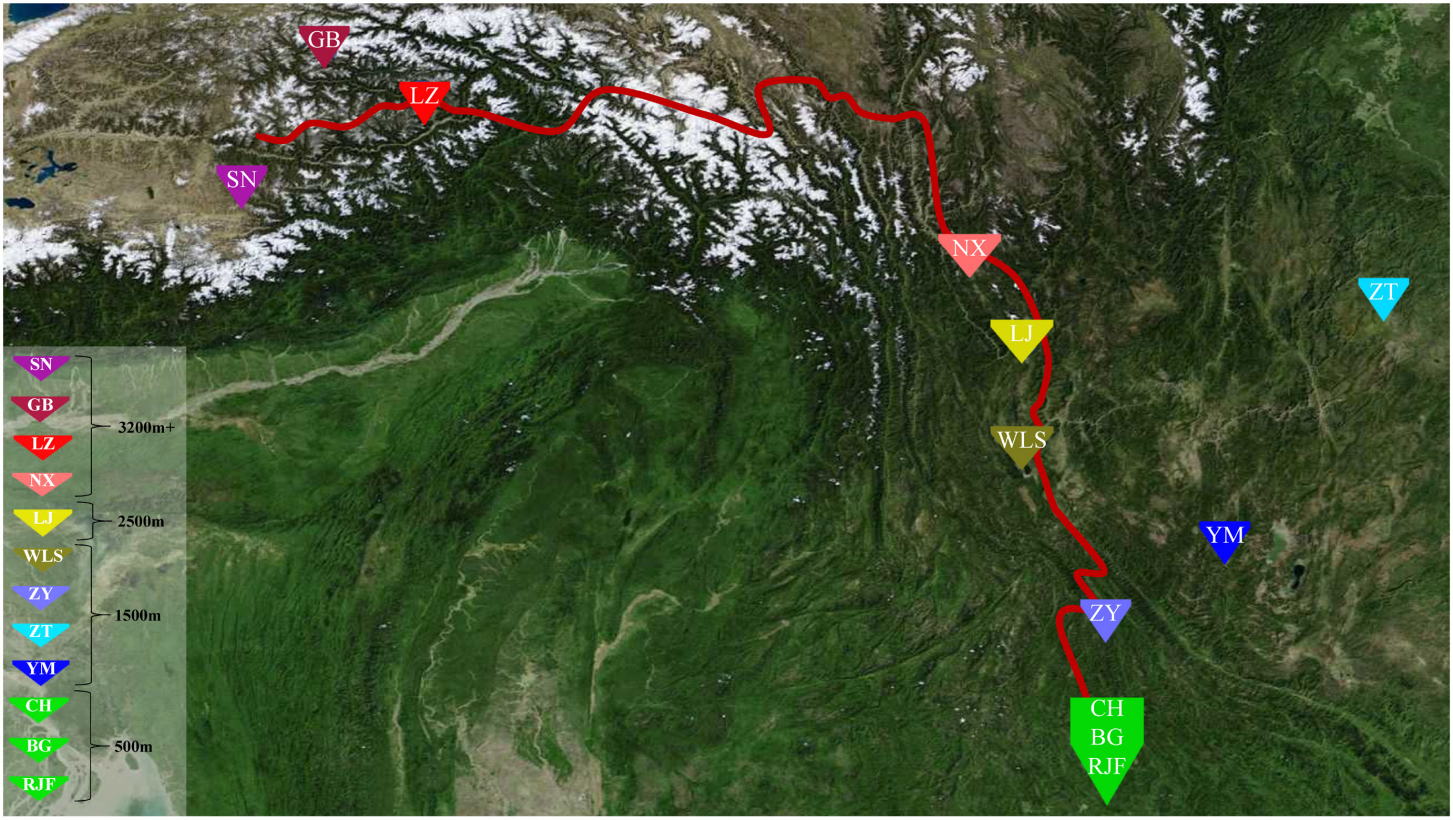
Locations of the sampled populations. Colored signs represent the geographic distribution of the sampling localities of 12 chicken populations including the Shannan (SN) chicken, Gongbujiangda (GB) chicken, Linzhi (LZ) chicken, Nixi (NX) chicken, Lijiang (LJ) chicken, Wuliangshan (WLS) chicken, Yimen (YM) chicken, Zhenyuan (ZY) chicken, Zhaotong (ZT) chicken, Chahua (CH) chickens, Banna game (BG) chicken, and red jungle fowl (RJF) from four altitudes (500, 1500, 2500, and 3200 m+). The red line presents the Ancient Tea Horse Road crossing Yunnan and Tibet.

One hundred and eighty‐eight chickens from 12 populations were collected from the area surrounding the Ancient Tea Horse Road (Figure 1), of which, 58 high‐altitude chickens (HACs) were from an altitude of above 3200 m, comprising 37 TBCs from Shannan (SN, 3600 m), Gongbujiangda (GB, 3600 m), and Linzhi (LZ, 3500 m) in the Tibet region and 21 Nixi chickens (NX, 3200 m) from the Yunnan region. An additional 21 chickens were from an altitude of approximately 2500 m, including Lijiang (LJ); 70 chickens were from an altitude of 1500 m, including Wuliangshan (WLS), Yimen (YM), Zhaotong (ZT), and Zhenyuan (ZY) sites, and 39 chickens were from an altitude of less than 500 m, including RJF, Banna game (BG) chickens, and Chahua (CH) chickens (Table S1). Thus, the samples represent four altitude levels, various geographic origins, and adaptation to high‐altitude environments. Two milliliters of blood were collected from a wing vein of all study chickens and stored at −20°C. DNA was extracted using the TIANamp Genomic DNA Kit (TIANGEN, Beijing, China) following the manufacturer's protocol.

### 2.2 Whole‐genome sequencing, reads mapping, and single‐nucleotide polymorphism (SNP) detection

Sequencing libraries were constructed using the TruSeq Nano DNA HT Sample Preparation Kit (Illumina Inc., San Diego, CA, USA) following the manufacturer's instructions, and index codes were added to attribute the sequences to each sample. In brief, DNA was fragmented to a size of 350 bp by sonication and end‐repaired, A‐tailed, and ligated to paired‐end adapter for sequencing with further polymerase chain reaction (PCR) amplification. The constructed libraries were sequenced on the Illumina HiSeq X Ten platform (Illumina Inc.) and paired‐end 150‐bp reads were generated. Sequence reads were filtered using fastp (v 0.12.3) (Chen et al., 2018), which discarded the reads of lengths <50 bp and N bases >6. The high‐quality trimmed read pairs were mapped on the reference genome GRCg6a (https://www.ncbi.nlm.nih.gov/assembly/GCF_000002315.6) using BWA‐MEM (v 0.7.17) (Li & Durbin, 2009), and the resulting alignment files were sorted using SAMtools (Li et al., 2009). The duplicated reads were marked using MarkDuplicates and base quality scores that estimated their biases were recalibrated using the BaseRecalibrator and ApplyBQSR command in GATK (v 4.1) (McKenna et al., 2010). SNPs were detected using the HaplotypeCaller command, and the output gVCF files were merged using the CombineGVCFs command. The SNPs were filtered using the VariantFiltration and SelectVariants commands with the following criteria: Quality (QUAL < 40.0), QualByDepth (QD < 2.0), FisherStrand (FS > 60.0), StrandOddsRatio (SOR > 10.0), RMSMappingQuality (MQ < 40.0), ReadPosRankSumTest (ReadPosRankSum < −8.0), MappingQualityRankSumTest (MQRankSum < −12.5), and CombinedDepth (DP < 5). Finally, we retained biallelic SNPs with max‐missing under 0.90 and a minor allele frequency of over 0.05 in autosomes. The dataset was further phased to impute its own missing positions using Shapeit (v 2.904) (Delaneau et al., 2011), and the SNPs were annotated using SnpEff (v 4.3 k) (Cingolani et al., 2012).

### 2.3 Genetic variation analysis

Linkage disequilibrium (LD) was calculated as the parameter *r* ^2^ between SNP pairs in VCF files with a maximum distance of 50 kb using PopLDdecay (v 3.4) (Zhang et al., 2019). We evaluated the genomic diversity for each breed and chicken population, including observed (*H*_o_) and expected heterozygosity (*H*_e_) as well as run of homozygosity (ROH) with a minimum length of 100 kb containing at least 50 SNPs and a maximum of 1 heterozygous variant using Plink (v 1.9) (Purcell et al., 2007). The nucleotide diversity (*θ*_*π*_) for each breed and population was calculated using a sliding‐window approach (100‐kb windows with 50‐kb steps).

### 2.4 Population phylogenetic and structure analysis

Principal component analysis (PCA) was performed using GCTA (v 1.91) (Yang et al., 2011) for the individual SNPs with the first three principal components. Plink (v 1.9) was used to transform VCF files and calculate pairwise identity‐by‐state (IBS) distance matrix data of all individuals. The neighbor‐joining (NJ) tree was constructed based on IBS using PHYLIP (v 3.695) (Felsenstein, 1989), and visualized using FigTree (v 1.4.3). Population structure was evaluated using ADMIXTURE (v 1.3) (Alexander et al., 2009), considering 2–8 genetic clusters (*K* = 2–8).

### 2.5 Migration events and admixture analysis

To investigate the migration and admixture of domestic chicken populations from the elevation gradient area, the separation and mixture graphs were inferred using TreeMix (v 1.13) (Pickrell & Pritchard, 2012) and qpGraph programs in ADMIXTOOLS (v 7.0.1) (Patterson et al., 2012). In these analyses, RJF and “500 m” were used as outgroups. Additionally, *f*3 (Reich et al., 2009) and *D*‐statistics (Durand et al., 2011) were used to measure introgression among HAC populations, which were computed using the threepop and qpDstat programs in TreeMix and ADMIXTOOLS, respectively.

### 2.6 Effective population size estimation and inference of demographic history

To estimate the effective population size, we applied SMC++ (v1.15.4) (Terhorst et al., 2017) under 1 year per generation time (Wang et al., 2021; Wang, Li, et al., 2015) and a mutation rate of 1.91 × 10^−9^ per site per year (Nam et al., 2010). To elucidate the demographic history of the TBC, we employed Fastsimcoal program (v 2.6) (Excoffier et al., 2013) and whole‐genome sequences to test four competing divergence pattern scenarios. We filtered sites within CpG islands, repeat‐masked regions, and protein genes and implemented LD pruning with Plink (v 1.9) using command “‐‐indep‐pairwise” (50 5 0.0001). This function calculates the pairwise LD estimate *r* ^2^ in a 50 SNPs window, shifts at a pace of 5 SNPs and excludes one of SNPs when *r* ^2^ > 0.0001. We then generated the SFS file using the python script vcf2sfs.py (https://github.com/marqueda/SFS‐scripts).

Owing to computational constraints and the correspondence of population phylogenetic and genetic structure analyses, we tested four alternative simplified models among populations from four different main geographic regions. In detail, eight populations were chosen, with SN, GB, and LZ representing the TBC populations and the NX and LJ representing the Jinsha River chickens (JR). WLS and ZY were used as the representative middle‐altitude region chickens (MA) and CH as the reference low‐altitude area chickens (LA). We assumed that the TBC was derived from the MA populations in model 1 and from the LA populations in model 2. In model 3, we considered that the JR was derived from the LA populations. The JR and TBC were both assumed to be derived from the MA populations in model 4. For each demographic model, we performed 500,000 simulations, 50 conditional maximization cycles, and 100 replicate runs starting from different random initial values. The best fit demographic model was identified by the Akaike information criterion (AIC, Akaike, 1974), and we re‐estimated the parameters using a nonparametric block‐bootstrap approach. In brief, 100 bootstrap data sets were obtained by dividing the SNPs into 100 blocks, and sampling with 100 replacement blocks for each bootstrap data set. The parameter estimates from the run with the highest likelihood from each bootstrap replicate with 10 replicate runs were then used to compute 95% confidence intervals with the R boot package.

### 2.7 Positive selection scans for the TBC

The TBC populations were chosen to analyze high‐altitude adaptation. We first used VCFtools (v 0.1.16) (Danecek et al., 2011) to calculate the population differentiation value between the TBC and “500 m” populations (CH, BG, and RJF) (*F*_st_; Weir & Cockerham, 1984) to scan the TBC genomes for signatures of positive selection with a 10‐kb sliding‐window and 5‐kb step size. In addition, we calculated the normalized XP‐nSL value (Szpiech et al., 2021) for each SNP based on each of the haplotype pools for each population using SELSCAN (v 1.3) (Szpiech & Hernandez, 2014) and performed sliding‐window analyses to calculate average XP‐nSL values with a window size of 10 kb and a step size of 5 kb. The thresholds to identify candidate genes in the *F*_st_ and XP‐nSL analyses were both set to the top 1% outliers, and Gene Ontology (GO) enrichment analysis was performed using DAVID (v 6.8) (Huang et al., 2009).

### 2.8 Detection of genetic variants associated with altitude

For the continuous altitudinal gradient model analysis, we applied Bayesian population association analysis (BayPass v 2.2; Gautier, 2015). We used four altitude levels as variables for eight populations in accordance with demographic analysis. Here, we applied the TBC and NX as the “3200 m+” population and LJ as the “2500 m” population; WLS, ZY, and CH represented the “1500 m” and “500 m” populations, respectively. The standard covariate model was used to perform a genome scan for differentiation by estimating the population covariance matrix of the allele frequencies. Functional candidate genes located within 20 kb upstream and downstream of the physical positions of these selected SNPs were identified based on the annotation of chicken reference. We also applied *F*_st_ using a sliding‐window approach with the same parameters mentioned above to calibrate and detect the variants selected by high altitude.

## 3 RESULTS

### 3.1 Genetic variations

We sequenced 188 whole genomes of Chinese native chickens belonging to 12 populations living at four continuous altitude levels. In total, approximately 1.51 T of raw sequence data was yielded, corresponding to genome coverage ranging from 13.68 to 15.55 (Table S2). After using strict read alignment and genotyping calling procedures, 12,971,498 autosomal SNPs were identified and used for further analysis (Figure S1), with a range of 8,300,160‐11,258,420 SNPs for each breed. Approximately 86% of the SNPs identified in our 188 Chinese native chickens were validated in the chicken dbSNP database. Following the SnpEff annotation, we found that the highest number of SNPs was located in the intronic region (6,281,060; 48.22%), followed by those in the intergenic (3,380,649; 26.06%), upstream (2,003,833; 15.45%) and downstream (574,042; 4.43%) regions (Table S3).

### 3.2 Linkage disequilibrium and patterns of genomic variation

Two breeds from the “3200 m+” (GB and LZ) altitudes showed an overall slow decay rate and a high level of LD, whereas the two other “3200 m+” (SN and NX) and “1500 m” (ZY and WLS) populations showed an overall fast decay rate (Figure S2). The heterozygosity analysis showed that SN, GB, NX, and LJ had smaller H_o_ than *H*_*e*_ (Table S4). The genome‐wide average *θ*_*π*_ values for the four altitude populations were similar, ranging from 3 × 10^−3^ to 4 × 10^−3^, whereas the RJF showed low *θ*_*π*_ values that might be due to the samples from a population with small family sizes (Figure S3). A larger average ROH size for chickens was observed in populations from the “500 m” altitude group. In particular, CH exhibited the largest average ROH. Other breeds such as SN and GB from the “3200 m+” group also exhibited a larger average ROH than other breeds from the “2500 m” and “1500 m” groups (Figure S4).

### 3.3 Phylogenetic relationships and population genetic structural analysis

**FIGURE 2:**
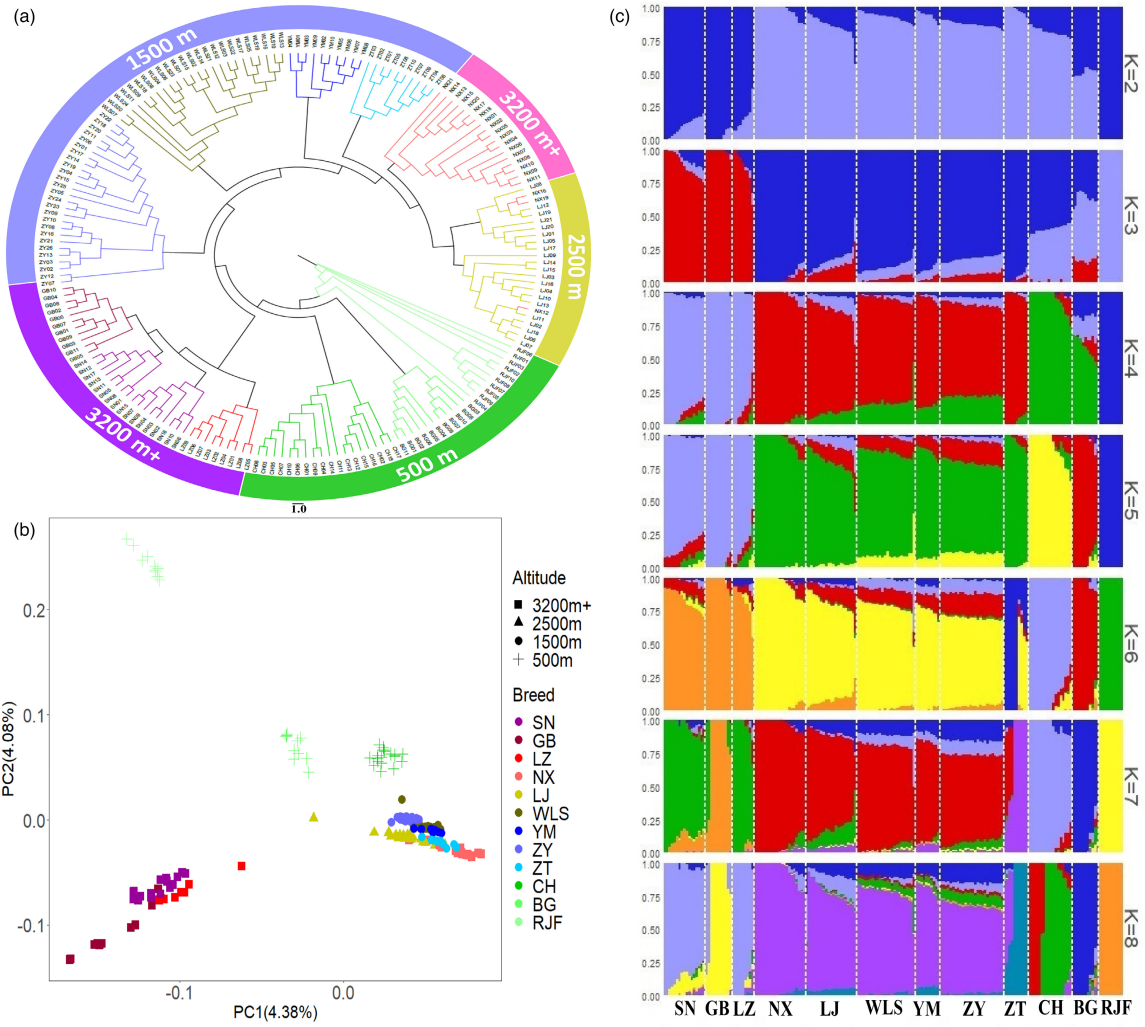
Population genetic structure of the native chicken in southwestern China. (a) Neighbor‐joining tree based on genome‐wide single‐nucleotide polymorphisms (SNPs) from four altitude groups. (b) Principal component analysis (PCA) of SNPs from chickens crossing gradient elevation areas. The plot is based on the first two PCAs. (c) Analysis of the genetic population structure of 188 Chinese native chickens was conducted using ADMIXTURE. The colors in each column represent the contribution from each subcluster (*K* = 2–8 when *K* = 6 has the lowest cross‐validation error). The chicken populations used included the Shannan (SN), Gongbujiangda (GB), Linzhi (LZ), Nixi (NX), Lijiang (LJ), Wuliangshan (WLS), Yimen (YM), Zhenyuan (ZY), Zhaotong (ZT), Chahua (CH), Banna game (BG) chicken, and Red jungle fowl (RJF).

To understand the phylogenetic relationships and population structure of the domestic chickens from the elevation gradient area, we utilized the whole‐genome SNP dataset of the 188 individuals. Using the RJF as an outgroup, the phylogenetic tree showed a distant relationship between the “3200 m+” (particularly SN, GB, and LZ) and “500 m” chickens, followed by “1500 m” chickens. The “3200 m+” chickens were split into two geographically structured clades. One is the TBC living in the Yarlung Zangbo River basin. Another is NX, which was grouped with the “2500 m” populations according to their geographical origin in the JR basin (Figure 2a). The PCA provided additional corroborating evidence, revealing that the chickens were clustered in several separate groups according to altitude. The first PCA axis separated the RJF and TBC from other chickens; both second and third PCA axes separated CH and BG from the “2500 m” and “1500 m” populations. The NX population living near the JR was always associated with populations living at altitudes of 2500 m and 1500 m (Figure 2b, Figure S5).

To determine the possible genetic admixture among the populations, we performed population structure analysis with a full maximum‐likelihood approach using ADMIXTURE, which estimates individual ancestry and admixture proportions assuming *K* ancestral populations. When *K* = 3, we observed a division between the RJF and TBC, as well as between the populations living at high and low altitudes. Besides, the RJF share a distinct genetic background with BG and CH based on *K* = 4 and 5. When *K* = 6, which is the best value exhibiting the lowest cross‐validation error, the result was largely consistent with the PCA results and supported that there were two divergent groups of HACs different from the lowland chicken; one group (NX) has a genetic makeup similar to that of Yunnan native chicken (Figures 2c, S6). Our results provide evidence that chicken breeds living at altitudes above 3200 m are genetically distinct from lowland chickens (500 m) and that HACs can be divided into two groups. These groups generally correspond to their geographic origins even though they both show high‐altitude adaptation.

### 3.4 Population admixture of the TBC

**FIGURE 3:**
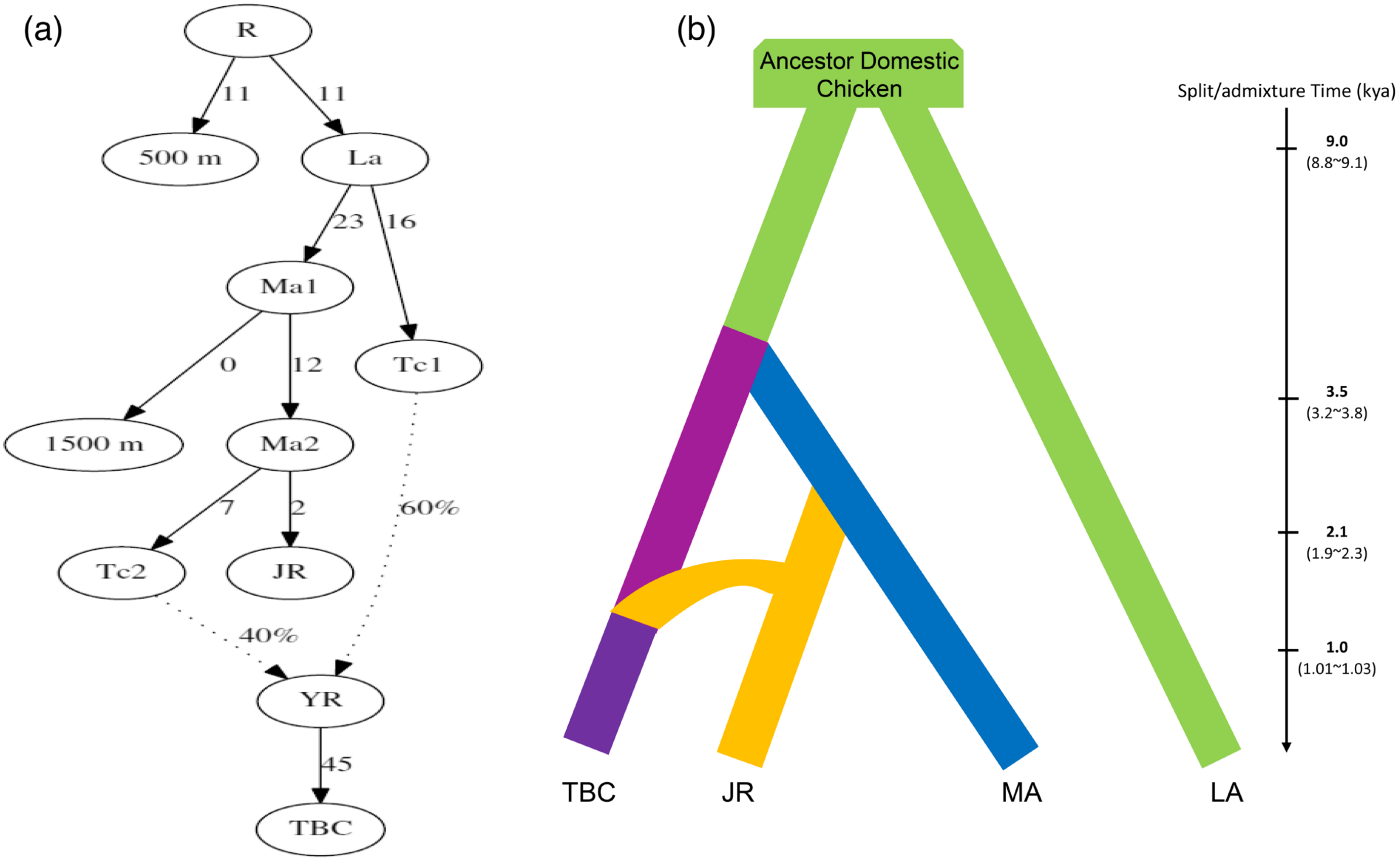
Modeling the history of two subgroups of the high‐altitude chicken (HAC). (a) Best fit model inferred using the qpGraph program for chicken populations from “1500 m” (Zhaotong [ZT], Zhenyuan [ZY], Yimen [YM], and Wuliangshan [WLS]), “JR” (Lijiang [LJ] and Nixi [NX] near the Jinsha River), and “TBC” (Linzhi [LZ], Gongbujiangda [GB], and Shannan [SN] near the Yarlung Zangbo River), using the “500 m” (Chahua [CH], Banna game chicken [BG], and red jungle fowl [RJF]) population as the outgroup. The branch lengths are shown in units of *F*_st_ × 1000. Dotted lines denote admixture events, and the values beside the dotted line correspond to admixture proportions. (b) Demographic history, including the divergence and migration times, for the Tibetan chicken (TBC), Jinsha River chicken (JR), middle‐altitude region chicken (MA), and low‐altitude area chicken (LA) inferred using the Fastsimcoal program.

The maximum likelihood (ML) tree without migration events inferred from the TreeMix analysis divided the HACs into two clusters (Figure S7), which are similar to the population structuring patterns identified from our analysis of the population genetic structure. Up to 99% of the variance between breeds was explained by a model with five migration events. In this model, we observed a migration edge from the JR (NX and LJ) to the TBC. In addition, we found that NX and chickens at an altitude of 1500 m had migration events, in line with the results of ADMIXTURE. To construct an admixture graph for four chicken populations from the altitude gradient area, we used the qpGraph program to build several models and used the “500 m” population as an outgroup. The model that best fit the data having no *f*4 outliers for chickens showed reasonable results. We found approximately two lineages in the best model. Firstly, the “1500 m” and “2500 m” populations were split in sequence. Another lineage showed the “3200 m+” population as an admixed population that had 54% ancestry derived from a lineage related to the “2500 m” population, and 46% ancestry from another diverged lineage (Figure S8). This is generally consistent with the result of TreeMix, supporting that the TBC had gene flow with the “2500 m” population. As our HACs could be divided into two subgroups, we adjusted our population to refine the admixture graph according to the genetic structure of the previous population. Interestingly, the best fit of qpGraph was generally consistent with the results of the model before subgroup adjustment, strongly suggesting that the TBC is an admixed population with the JR population (Figure 3a).

Further admixture analyses, the *f*3 test, and *D*‐statistics were performed. The *f*3 test indicated that the TBC carries more genetic ancestry from NX (higher *f*3 values) than other chicken breeds (Figure S9). *D*‐statistics showed significant values (|*Z*| > 3) with positive *D* values, suggesting a closer relationship between the TBC and NX chickens than between other chicken breeds, including the LJ breed that has a closer geographical location and genetic structure according to previous results (Figure S10). Our admixture analysis results showed evidence of gene flow between the subgroups of HACs and introgression of NX ancestry in the TBC; which is in line with the TreeMix and qpGraph findings.

### 3.5 Demographic history of the TBC

The SMC++ analysis showed that the population size of the chicken ancestor increased approximately one million years ago, reached a peak approximately 100 thousand years ago, and subsequently experienced a substantial decrease until approximately 10,000 years ago (Figure S11). These findings are consistent with previous study results (Wang, Cheng, & Schmid, 2015). According to a previous study on the origin and domestication of chickens (Wang et al., 2020) and our admixture analysis, we compared four alternative demographic models to rigorously infer the most probable divergence pattern and migration route of the two subgroups of HACs (Figure S12). A comparison of AIC showed that model 2 had the lowest AIC among the four models (Figure S13), supporting the qpGraph results. The best‐supported model 2 indicated a one‐step divergence pattern and a southwest‐to‐northwest migration route for the HACs. That is, the chicken ancestors that inhabited an altitude of 500 m were domesticated, the LA chicken populations were formed approximately 9000 years ago, and they later migrated to a higher altitude where they formed the MA chicken population, as well as an ancient Tibetan group in the Tibetan plateau approximately 3500 years ago. The MA chicken population subsequently migrated northwest and generated the JR chicken population approximately 2100 years ago. Finally, the JR chicken population further migrated to the Tibetan plateau and admixed with the original Tibetan population over a 1000 years, which finally became the TBC population (Figure 3b).

### 3.6 Selection signatures in the TBC

**FIGURE 4:**
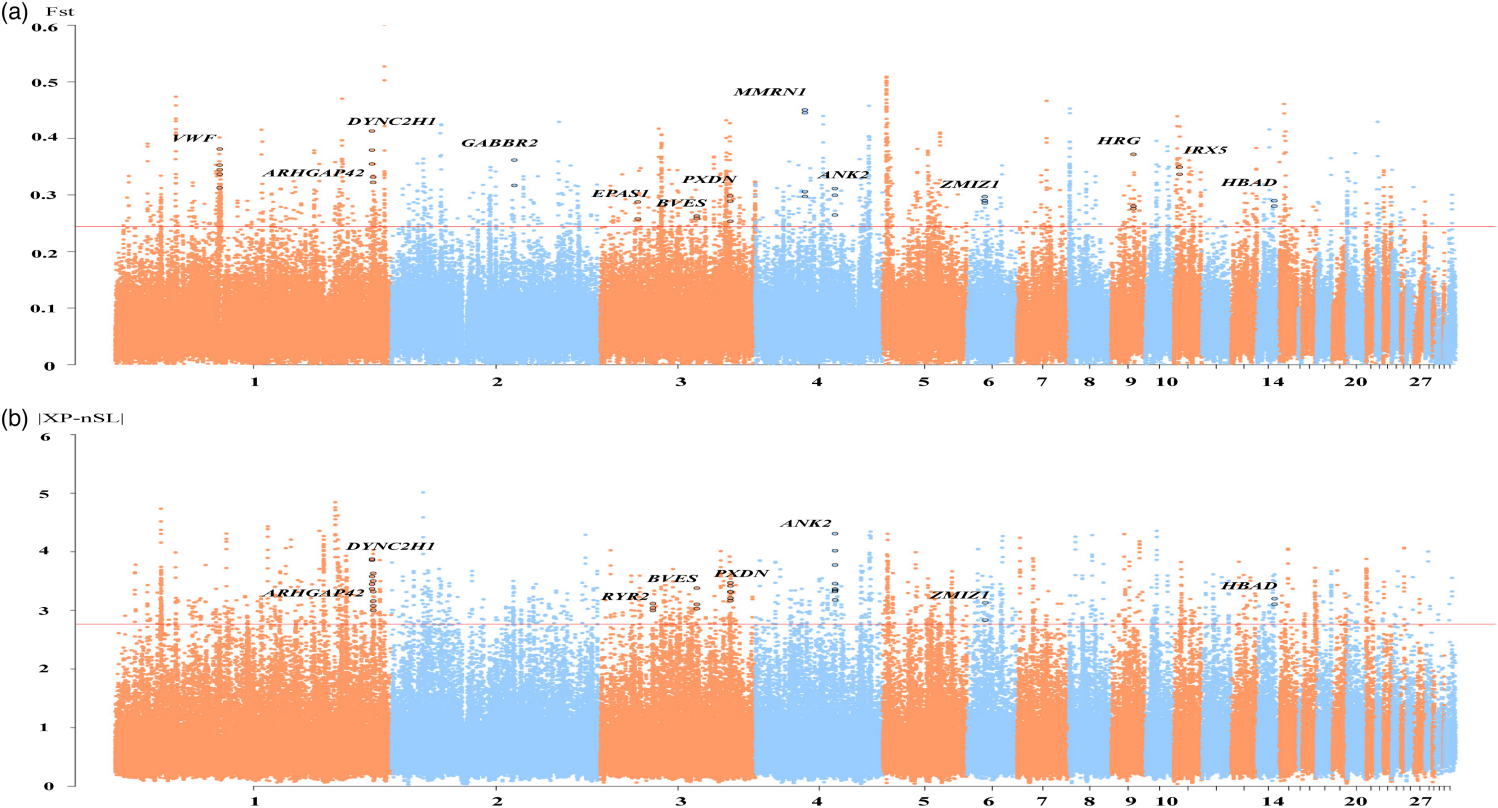
Positive selection scans for adaptation to high altitude. Red line, selection criteria for each test; black dots, strongly selected windows or single‐nucleotide polymorphisms (SNPs). (a) *F*_st_ value as calculated by comparing the TBC and “500 m” groups. (b) Normalized absolute XP‐nSL value is shown by comparing the TBC and “500 m” groups.

In positive selection scans between the “500 m” (CH, BG, and RJF) and TBC populations, the top 1% *F*_st_ (*F*_st_ ≥0.24) contained 1860 genomic regions and comprised 399 candidate genes (Figure 4a, Table S5). Among the candidate genes, we found that some genes and pathways may be associated with hypoxia. Nine genes (*DPP4*, *EGR1*, *BECN1*, *MYOCD*, *SCFD1*, *EPAS1*, *CASP3*, *CXCR4*, and *KCNA5*) are involved in the response to hypoxia, three (*HBZ*, *HBAD*, and *HBA1*) are involved in oxygen transport, three (*DOCK5*, *ARHGAP42*, and *DOCK4*) are enriched in negative regulation of vascular smooth muscle contraction, and four (*IRX5*, *EPAS1*, *ANK2*, and *BVES*) are involved in regulation of heart rate. In addition, we noted that five genes (*MMRN1*, *VWF*, *ALB*, *HRG*, and *KNG1*) are involved in platelet alpha granule lumen, which could affect platelet degranulation and blood coagulation (Table S6). We also used the *F*_st_ analysis between the TBC and other altitude populations (“1500 m” and “2500 m”). The selection signatures were incompatible, which might be due to different selective pressures between the groups (Figure S14).

Meanwhile, we applied a novel haplotype‐based scan for local adaptation (XP‐nSL), which has the power to detect ongoing and recently completed hard and soft sweeps, and then applied this statistic to search for evidence of high‐altitude adaptation in chickens. Moreover, as the positive and negative values of XP‐nSL indicate the directionality of selection, all SNPs within an XP‐nSL value are needed to show the same directionality. The normalized top 1% absolute XP‐nSL values corresponded to longer and higher frequency haplotypes and were located in 462 genes (Table S7). To further investigate the potential adaptation to high altitude, we overlapped the results of *F*_st_ and XP‐nSL and detected 117 genes, providing strong evidence for the adaptive role of high‐altitude adaptation (Table S8). Among the candidate genes obtained from XP‐nSL, we highlighted a list of genes (e.g., *HBAD*, *ANK2*, *BVES*, *ARHGAP42*, *ZMIZ1*, *DYNC2H1*, and *PXDN*) that were highly selected in *F*_st_, and *RYR2* with high positive selection, providing further insight into the molecular mechanisms underlying the high‐altitude hypoxia adaptation of the TBC (Figure 4b).

### 3.7 Genomic selective signatures by altitudinal gradient chicken population

**FIGURE 5:**
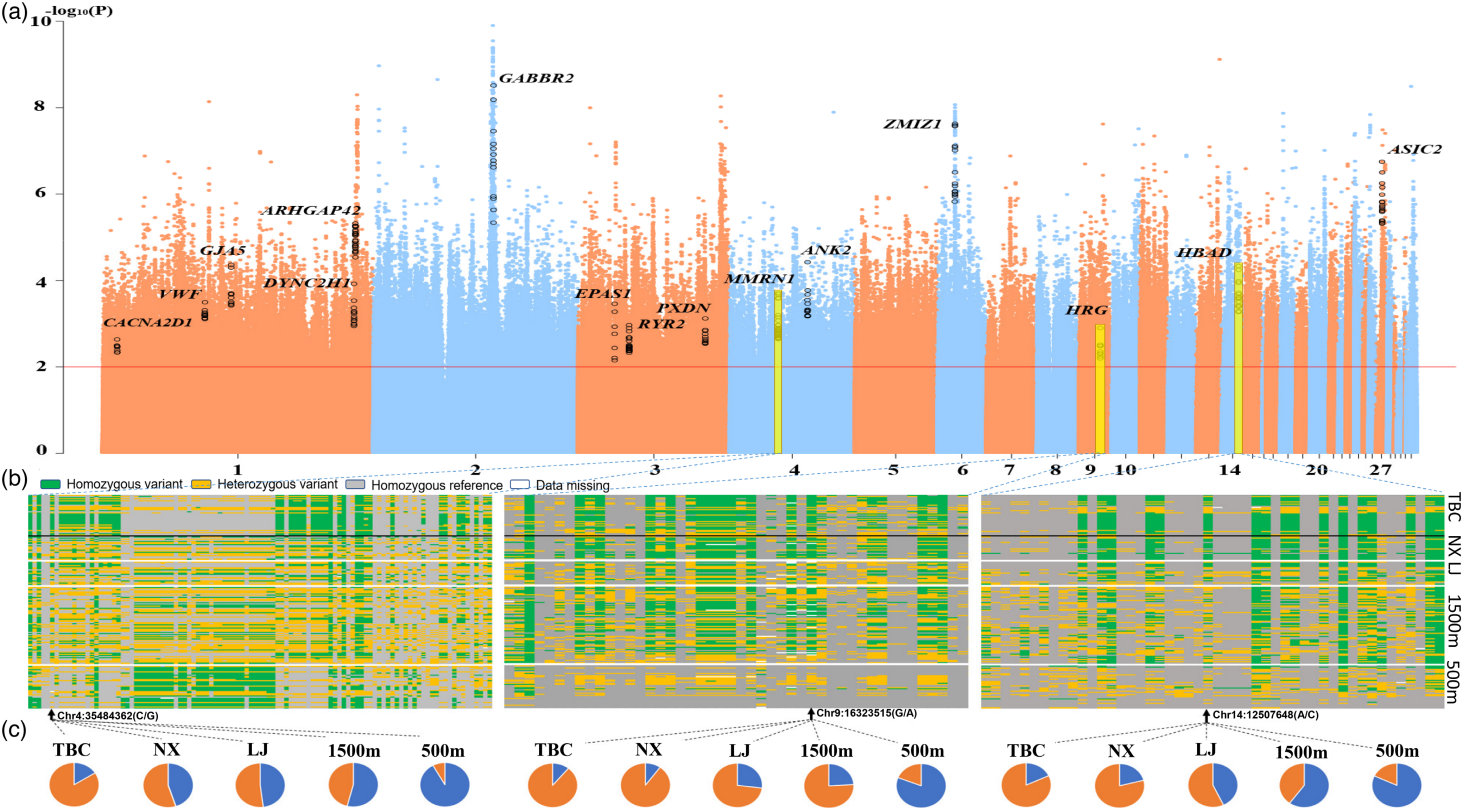
Detection of altitude‐related genetic variants using BayPass. (a) Genomic landscape of the *p*‐values in the BayPass analysis in the genomes of domestic chickens at four altitude levels. Red line, selection criteria for BayPass; black dots, strongly selected single‐nucleotide polymorphisms (SNPs). (b) Pattern of genotypes among chickens at all altitudes in the selected genomic region. (c) Pie charts representing the spectrum of allele frequencies at the missense loci of the focused genes *MMRN1*, *HRG*, and *HBAD* (from left to right) in the Tibetan chicken (TBC), as well as Nixi (NX), Lijiang (LJ), and other chickens inhabiting 1500 m and 500 m altitudes. The type of variant allele is indicated in orange, whereas the reference allele is indicated in blue.

To gain genomic selective signatures at the SNP level, we exclusively used the XtXst value calculated by BayPass. Here, we used a corresponding *p*‐value <0.01 as the selection criterion and obtained 249,613 selected variants (Figure 5a, Table S9). Similar to the method for detecting candidate genes in the TBC by *F*_st_, we detected candidate genes in NX (Figure S15, Table S10). Later, we combined the results of BayPass and win‐*F*_st_ from the TBC and NX. Thus, we found 716 selective genes (Table S11). Here, we not only identified genes in *F*_st_ (e.g., *DYNC2H1*, *ARHGAP42*, *HBAD*, *MMRN1*, *HRG*, *GABBR2*, *PXDN*, and *ZMIZ1*), but also novel genes, such as *GJA5*, *ASIC2*, and *CACNA2D1*. The overlapping genes were primarily related to the GO terms regulation of heart rate, negative regulation of vascular smooth muscle contraction, artery morphogenesis, and regulation of ventricular cardiac muscle cell membrane repolarization (Table S12). Further analysis showed that *MMRN1*, *HRG*, and *HBAD* contain missense mutations, which were selected using BayPass with gradient frequency changes according to the altitude gradient (Figure 5b,c).

## 4 DISCUSSION

In this study, we collected large‐scale genomic data for chicken populations from an elevation gradient and reconstructed the population history of the TBC. By characterizing the positive selected genes, we identified several molecular mechanisms that were likely contributors to the high‐altitude adaptation of the TBC.

The genomic variations analyses, such as ROH and LD, implied that the chickens living in Tibet generally had a high genetic diversity, which may be due to a lack of artificial selection. The genetic landscape matched the geographic subdivisions, showing a lowland chicken group and the TBC group and revealed that the HACs were divided into two genetically distinct clusters, even if they lived at a high altitude: one located in Tibet near the Yarlung Zangbo River, the other in Yunnan near the Jinsha River. Besides, we noticed that chickens that lived near the Jinsha River have had a similar genetic makeup to the chicken population at 1500 m. Overall, this could suggest that chickens in Yunnan and Tibet have been derived from different populations for a long time.

Reconstruction of the history of the HAC populations by demographic modeling suggested that the TBC and LA diverged 3.2–3.8 kya with subsequent admixture from the JR 1.01–1.03 kya. Our results were consistent with that of previous admixture analyses, suggesting that chickens in Yunnan had migrated to Tibet. The Ancient Tea Horse Road would appear to have been in use long before it became an avenue for the tea and horse trade during the Tang and the Song dynasties, as it was a very important corridor connecting the ancient cultures of Tibet and Yunnan (Yang, 2004). We infer that this historic road might have played a role in the evolution and admixture of the TBC, as well as the migration of the Yunnan chicken populations. Taken together, the phylogenetic and demographic analyses revealed genetic structure differences among the domestic chickens that inhabit different altitudes, as well as the evolution of the two subgroups of HAC at different times.

As our HACs have two subgroups with the same altitude adaptation but different genetic backgrounds, we applied several methods to explore the mechanism of high‐altitude adaptation in the TBC. We identified several candidate genes for the TBC subgroup using the *F*_st_ analysis combined with XP‐nSL. We observed that *HBAD*, and the frequency of its missense mutation, increased the oxygen affinity with increased altitude. Interestingly we noticed the same A94C (chr14:12507648) mutation in *HBAD* which is present in both the TBC and NX; this mutation may increase the oxygen affinity of hemoglobin owing to the lower hydrophobicity of leucine than that of methionine, potentially stabilizing the hemoglobin oxygen structure (Gou et al., 2007). In contrast to the hypoxia‐induced reduction in hemoglobin‐oxygen affinity that is typical of humans and other lowland mammals, many high‐altitude vertebrates have evolved genetically based increases in hemoglobin‐oxygen affinity in comparison with lowland relatives, and increased hemoglobin‐oxygen affinity is beneficial in severe hypoxia (Storz & Bautista, 2022). Based on our result, we think genetic polymorphism in hemoglobin is an important implication for the evolutionary dynamics in high‐altitude populations. Notably, *EPAS1*, which encodes hypoxia‐inducible factor‐2α (HIF‐2α), is also selected with high *F*_st_ values. Population genomic researches are useful for generating information about the genetic basis of high‐altitude adaptation. In Tibetan highlanders, one of the most extreme environments dwellers, such genome scans have implicated central components of the HIF signaling pathway, which orchestrates the transcriptional response to hypoxia (Beall et al., 2010; Peng et al., 2017; Simonson et al., 2010; Yang et al., 2017; Yi et al., 2010). Members of the HIF family of transcription factors exert oxygen‐dependent control over the tissue‐specific expression of target genes and regulate physiological response to hypoxia, including respiration, blood flow, vascular remodeling, and cardiac function (Peng et al., 2006; Samanta et al., 2017; Semenza, 2012, 2014). According to previous studies, increased hemoglobin concentration after exposure to high‐altitude conditions has negative effects on individuals from lowlands, and *EPAS1* is a well‐known gene under positive selection in Tibetan and Tibetan domestic mammals, which can attenuate maladaptive physiological alterations such as elevated hemoglobin concentrations (Peng et al., 2017; Wu et al., 2020). We assume that *EPAS1* can also play a similar role in the TBC. Besides, *RYR2* was detected using BayPass and XP‐nSL in the TBC, which was also reported in a previous study on the TBC (Wang, Li, et al., 2015); it is involved in cardiac muscle hypertrophy and heart rate regulation (Søndergaard et al., 2020; Yamaguchi et al., 2007).

According to a previous study, changes in cardiovascular system components, such as the heart and chorioallantoic membrane, are crucial for high‐altitude adaptation for the TBC (Zhang & Burggren, 2012). Besides, acute hypoxia can activate the sympathetic nervous system, which affects the heart rate, systemic vascular resistance, and blood pressure (Hainsworth & Drinkhill, 2007). Here we identified some novel genes (*ARHGAP42*, *ANK2*, *IRX5*, *DYNC2H1*, and *BVES*) that reportedly play roles in the regulation of vascular smooth muscle contraction (Bai et al., 2013), heart rate (Al Sayed et al., 2021; Roberts et al., 2019), heart development (Li et al., 2015), and ventricular outflow tract (Shi et al., 2020). We also identified some novel genes that may be highly related to high‐altitude adaptation with high selection. For example, we found that *GABBR2*, which encodes a subclass of receptors to regulate the Ca^2+^ concentration (Wang, Li, et al., 2015) and plays an essential role in preventing ischemic damage to tissue (Zhang et al., 2021), was highly selected. Here, we detected *ZMIZ1*, which plays a crucial role in vasculogenesis and heart morphogenesis (Beliakoff et al., 2008) and may contain super‐enhancer SNPs that are associated with coronary artery disease (Gong et al., 2018). In addition, cardiac fibrosis can be induced by hypoxic conditions (Watson et al., 2014). Here, we detected *PXDN*, a gene primarily expressed in the cardiovascular system and reported to be a regulator of cardiac fibrosis (Liu, Zhang, et al., 2019). Information of these genes may provide insights regarding the functional changes in the cardiovascular system that facilitate high‐altitude adaptation in the TBC. Similarly, high‐altitude mammals and birds exhibit a characteristic suite of derived changes in respiratory and cardiovascular traits compared to their closest low‐altitude relatives (Ivy & Scott, 2015; McClelland & Scott, 2019; Schweizer et al., 2019; Storz & Scott, 2019). To deal with the reduced availability of oxygen at high altitude, vertebrates in high altitude have evolved myriad adjustments and enhancements in the cardiorespiratory system and aerobic performance to match tissue oxygen delivery with metabolic oxygen demand (Storz & Scott, 2019). Hence, genetic effects on physiological traits of hypoxia adaptation in high‐altitude vertebrates are crucial and needed to be understood deeply.

Exposure to subacute hypoxic conditions can increase whole blood coagulation. Hypoxia is a stimulus for thrombus formation, and hypoxia‐inducible factors control the vascular response to hypoxia (Gupta et al., 2019). Notably, we identified novel genes *MMRN1*, *VWF,* and *HRG* that showed a strong selective sweep in the TBC. *MMRN1* can bind to the protein region encoded by another selected gene, *VWF*, to support platelet adhesion (Parker et al., 2016); this gene is involved in blood coagulation, and we assumed that the missense mutation in *MMRN1* may affect this biological process. Simultaneously, we noticed that *HRG*, which participates in the regulation of angiogenesis, coagulation, and fibrinolysis (Poon et al., 2011), was also highly selected. Specifically, *HRG* can modulate the intrinsic coagulation pathway by binding to factor XIIa and abrogate nucleic acid‐driven coagulation, serving as a novel modulator (MacQuarrie et al., 2011; Vu et al., 2015). Therefore, the highly continuous missense variants (chr9:16323515) may lead to understanding the function of *HRG* in the TBC. We suppose that traits of blood coagulation in high‐altitude animals may be beneficial because hypoxia environment could induce viscosity‐related impairments of cardiac function and microcirculatory blood flow (Storz, 2021), and our findings suggest the changes of blood coagulation can play crucial roles in high‐altitude adaptation for highland populations.

As for NX, we identified *ASIC2*, which was not highly selected in the TBC. This gene is an important determinant of autonomic circulatory control and baroreceptor sensitivity (Lu et al., 2009). Other genes, such as *CACNA2D1* and *GJA5*, which are involved in regulation of heart rate by the cardiac conduction system (Bourdin et al., 2015) and play a functional role in flow‐driven arteriogenesis (Buschmann et al., 2010), were also highly selected only in NX. However, this result may be attributed to their different origins and habitats. Although the two HAC subgroups originated from distinct breeds at different time points, with certain candidates that were found to have slight differences, they share some selective signatures in genes such as *HBAD*, *ZMIZ1*, *DYNC2H1*, *HRG*, *ARHGAP42*, and *PXDN*. The genotype frequencies of certain SNPs in the selective signature may be associated with phenotype of plateau adaptability in chickens. For instance, the mutation of A94C (chr14:12507648) in *HBAD* can increase the oxygen affinity of hemoglobin and facilitate hypoxic adaptation of chickens (Gou et al., 2007). The selective sites which can be applied as molecular markers are used in breeding to improve plateau adaptability for high‐altitude chicken populations. The traits of plateau adaptability, which reflect in eggs hatchability, surviving rate, and growth rate in chickens under high‐altitude environment, are crucial for plateau chicken industry (Wu & Li, 2012). Therefore, we believe our results can help to increase the breeding population size of Tibetan chicken.

**FIGURE 6:**
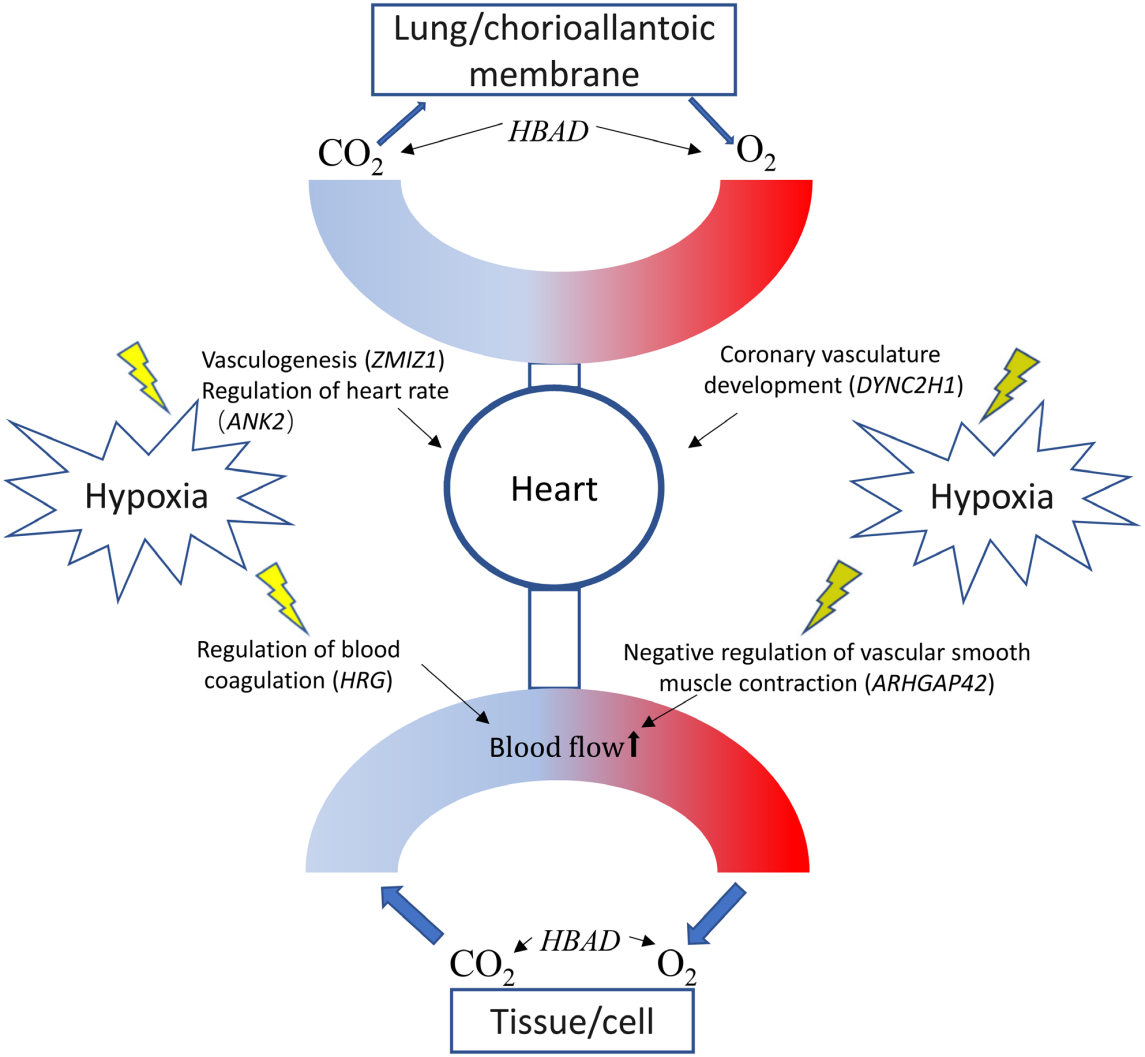
Functions of the key positively selected genes in biological processes for adaptation to high altitude.

Although some studies have reported that genes associated with high‐altitude adaptation in the TBC are mainly involved in the calcium‐signaling pathway, energy metabolism, body size maintenance, and regulation of gene expression (Li et al., 2019; Liu et al., 2020; Wang, Li, et al., 2015), our results demonstrate that the changes in the oxygen transport pathway and heart and blood vessel development in the cardiovascular system are crucial under hypoxic conditions (Figure 6). The results of our study are unique for a variety of reasons. First, we studied populations from maximum and minimum altitudes using the extreme adaptation gradient combined with continuous elevation gradients, which aids in the selection of genetic variants that are highly linked with the environment. This “two‐gradient” system could directly detect variants associated with high‐altitude environments. Second, the individual chickens from the QTP are widespread and numerous and could be highly representative of the HAC population and good resources to research high‐altitude adaptation. In studies of environmental adaptation, documenting an association between genotype and phenotype represents an essential step that can guide the hypotheses about causal mechanisms (Storz, 2021). We believe our results which are mainly associated with oxygen transport as well as cardiovascular system involved in physiological response to hypoxia can give clues to guide the design of follow‐up experiments to test hypotheses about causal mechanisms for highlanders and high‐altitude animals.

In conclusion, our findings demonstrated the genomic variability, population structure, and demographic history of the TBC and revealed a variety of well‐known and novel genes, as well as important GO categories that are associated with the high‐altitude adaptation of chickens. Specifically, the gradient altitudinal model analysis revealed that the candidate genes and GO terms were functionally related to hypoxia responses, oxygen delivery, and cardiovascular system function in the plateau environment. These results lay a foundation to elucidate the mechanisms of high‐altitude adaptation in chickens and other livestock species, particularly avian species, to facilitate their survival in the QTP. Furthermore, these newly generated genome‐wide data are valuable resources for future genetic improvement of the TBC.

## CONFLICT OF INTEREST

The authors declare that they have no conflict of interest.

## Supporting information


Figure S1

Table S1

## Data Availability

Raw sequence reads are deposited in the Sequence Read Archive (SRA) database (BioProject PRJNA782225).
