# Supplementary material for: Microevolutionary mechanism of high‐altitude adaptation in Tibetan chicken populations from an elevation gradient
Source: Evol Appl. 2022 Oct 31;15(12):2100–12. doi: 10.1111/eva.13503 (PMC9753841; doi:10.1111/eva.13503)

Supplementary Figure for

Microevolutionary mechanism of high-altitude adaptation in Tibetan chicken populations from an elevation gradient

Figure S1. Number of SNPs within a 20-k window across the chicken genome.

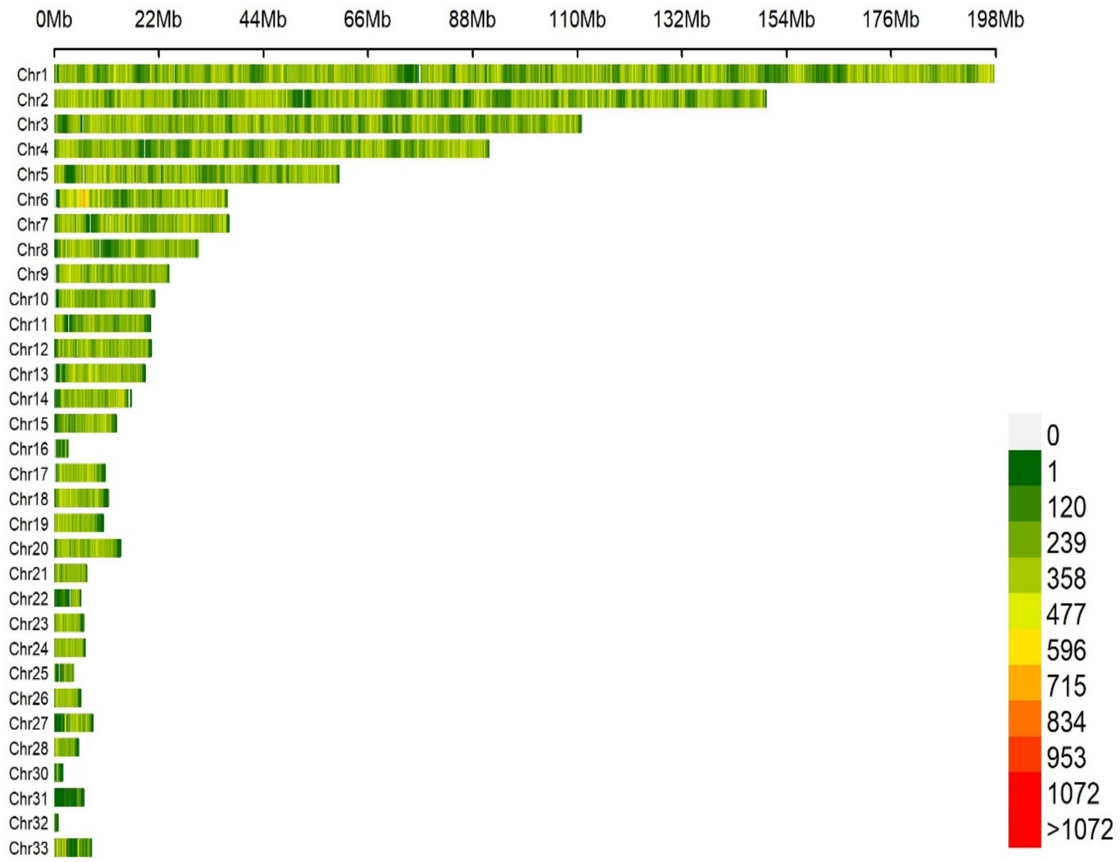

Figure S2. Decay of linkage disequilibrium (LD) in the chicken genome for each breed and population.

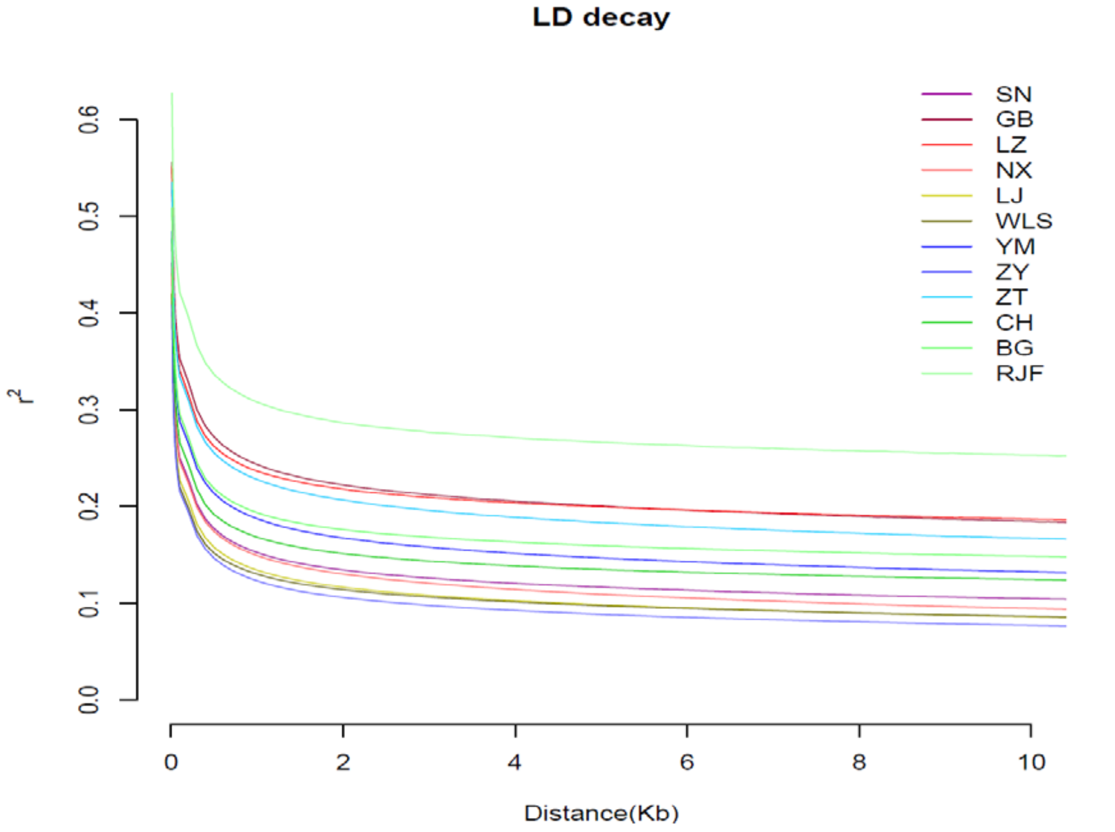

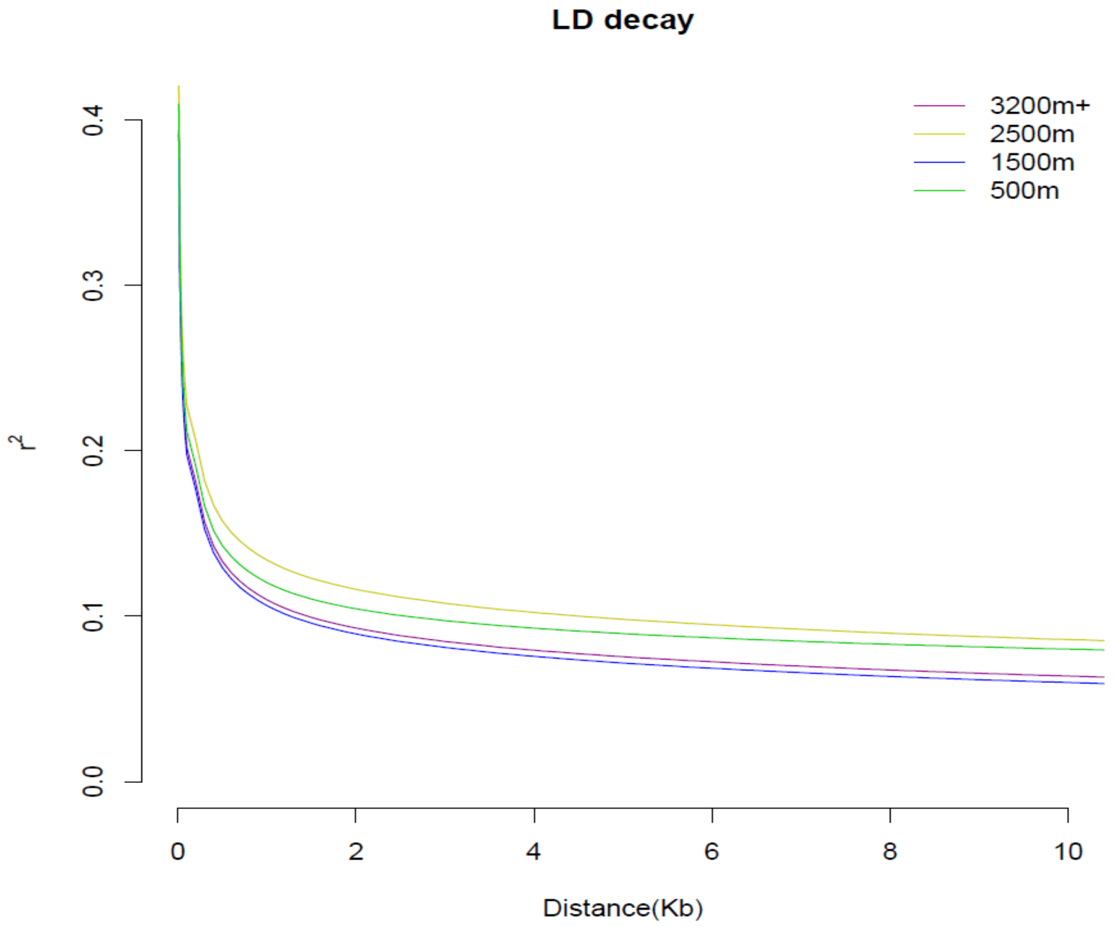

Figure S3. Breed and population average pairwise nucleotide diversity (θ_π) in the genomes of Chinese native chickens.

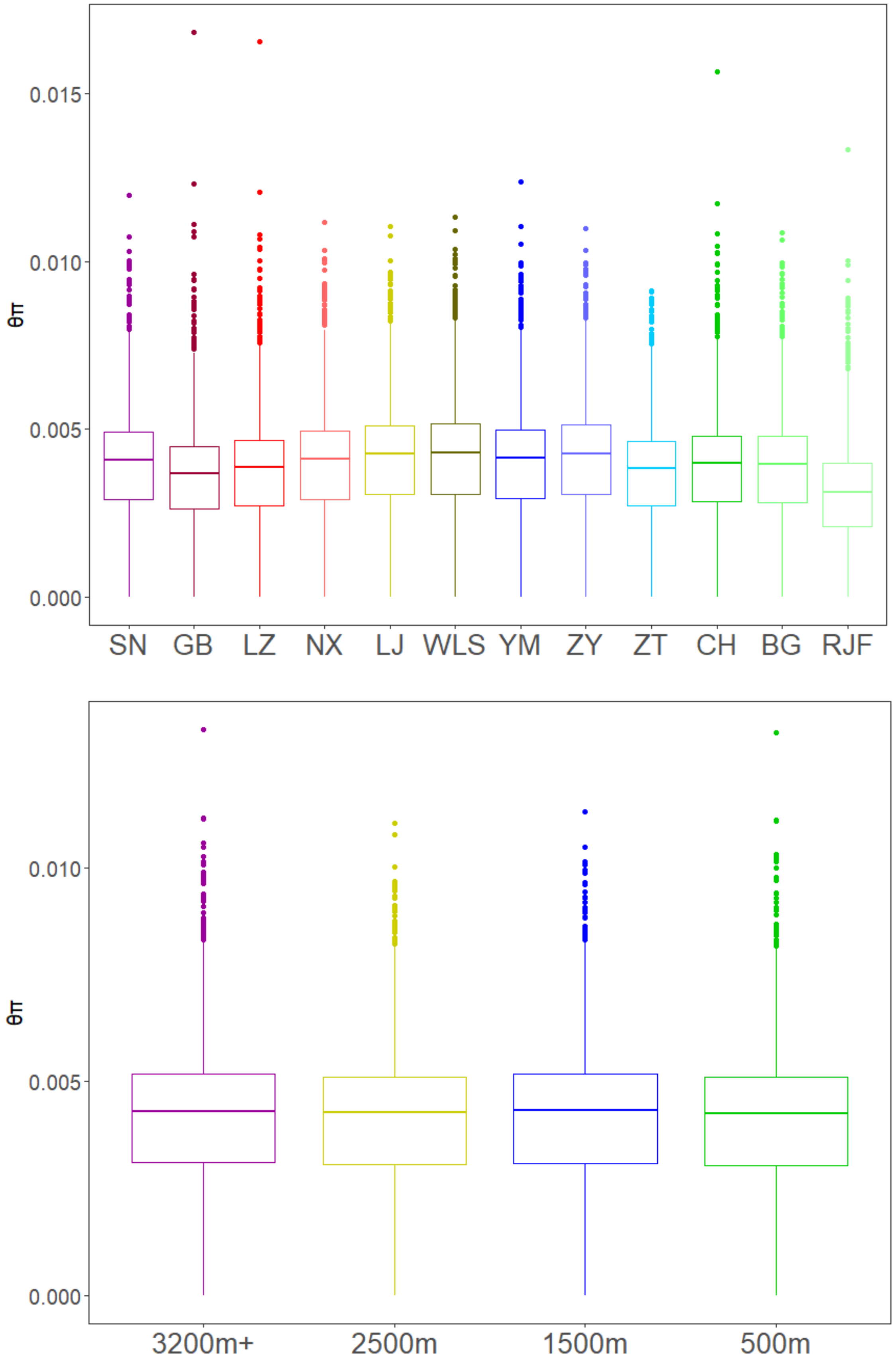

Figure S4. Average regions of homozygosity (ROH) size in the genomes of each Chinese native chicken breed and population.

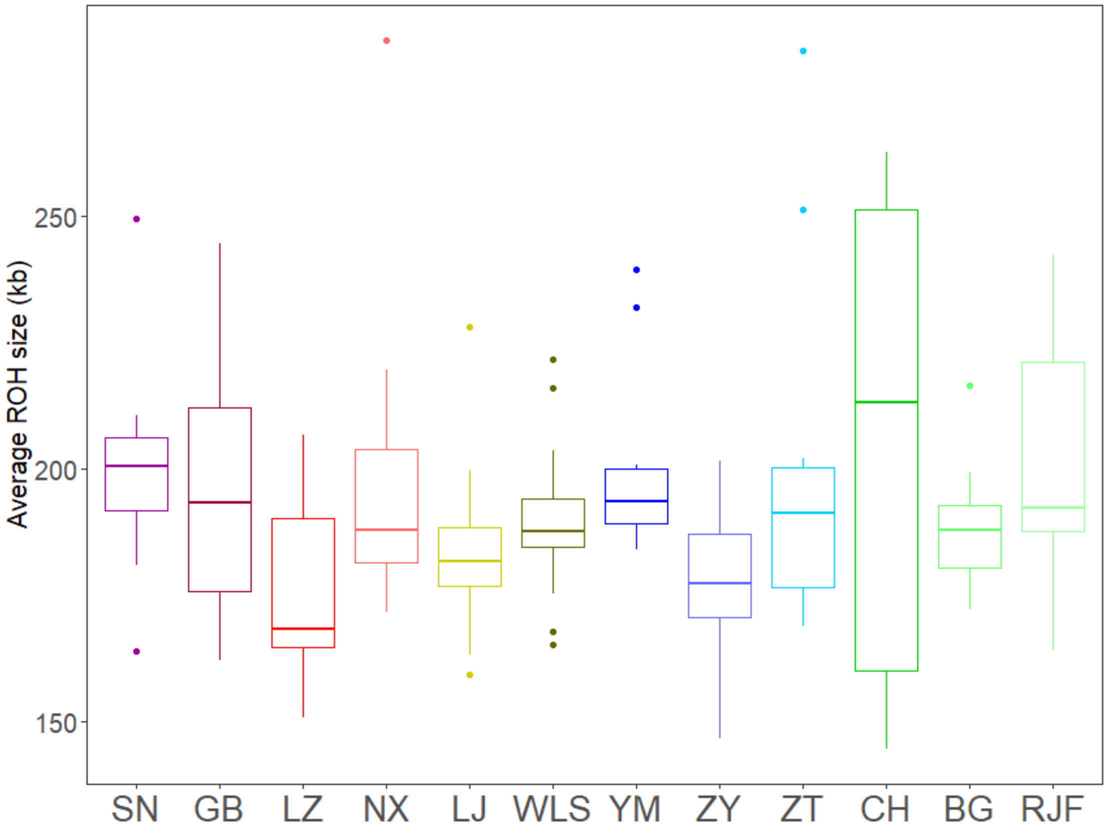

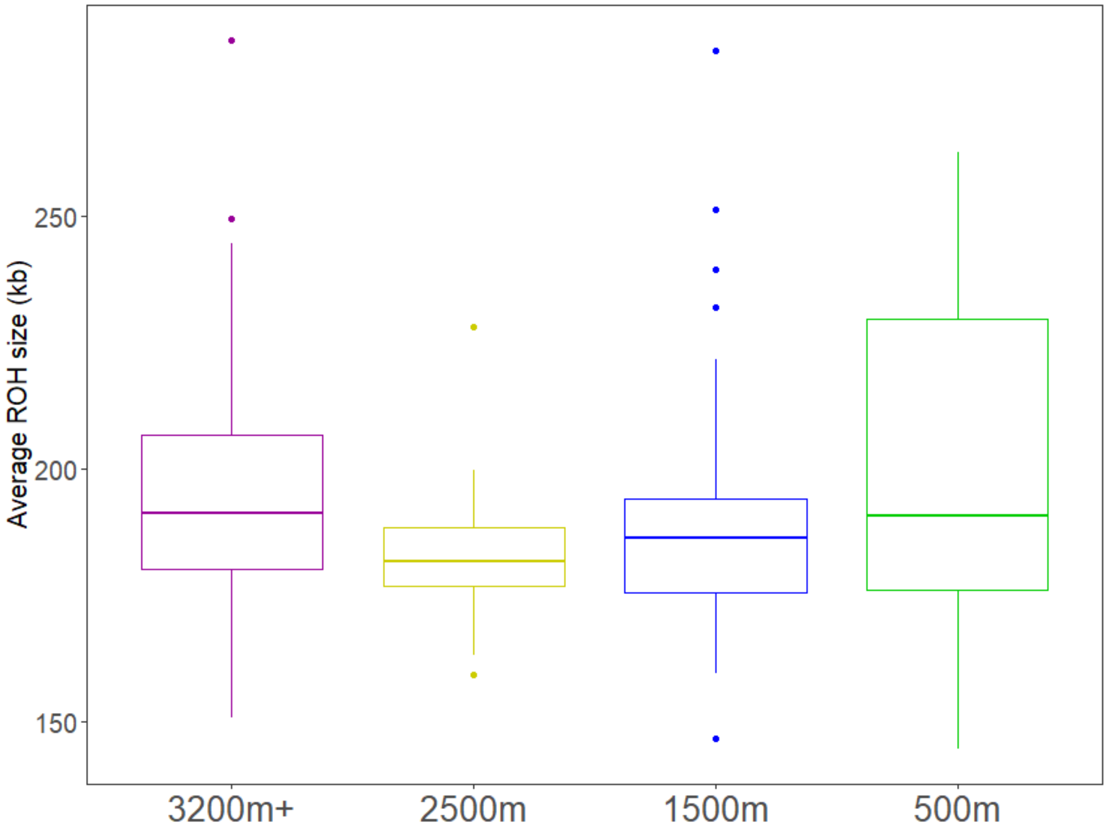

Figure S5. Principle component analysis (PCA) results for the first and third components of 188 chickens.

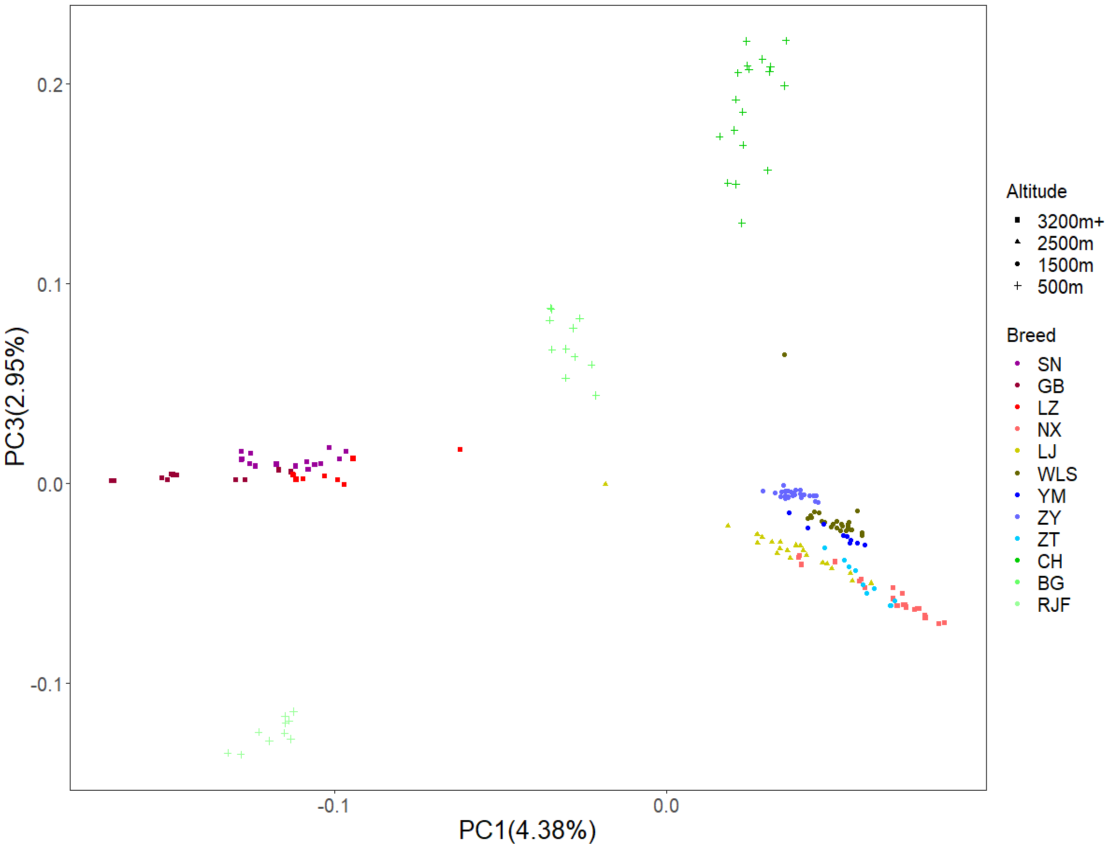

Figure S6. Cross-validation error for ADMIXTURE analysis.

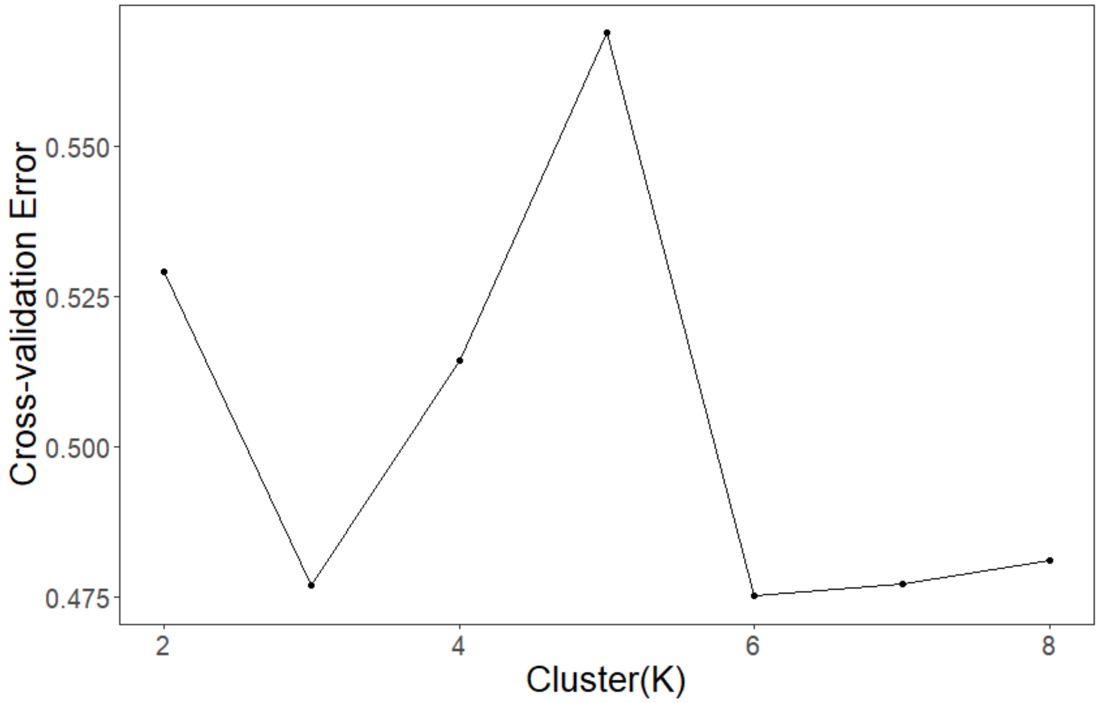

Figure S7. Migration analysis of chicken breeds using TreeMix software. (a) M=0-5 panels represent models of population affinities assuming 0-5 migration edges in TreeMix. The inferred migration weight is provided by the color of the arrow displayed. (b) Residual fit for models M=0-5.

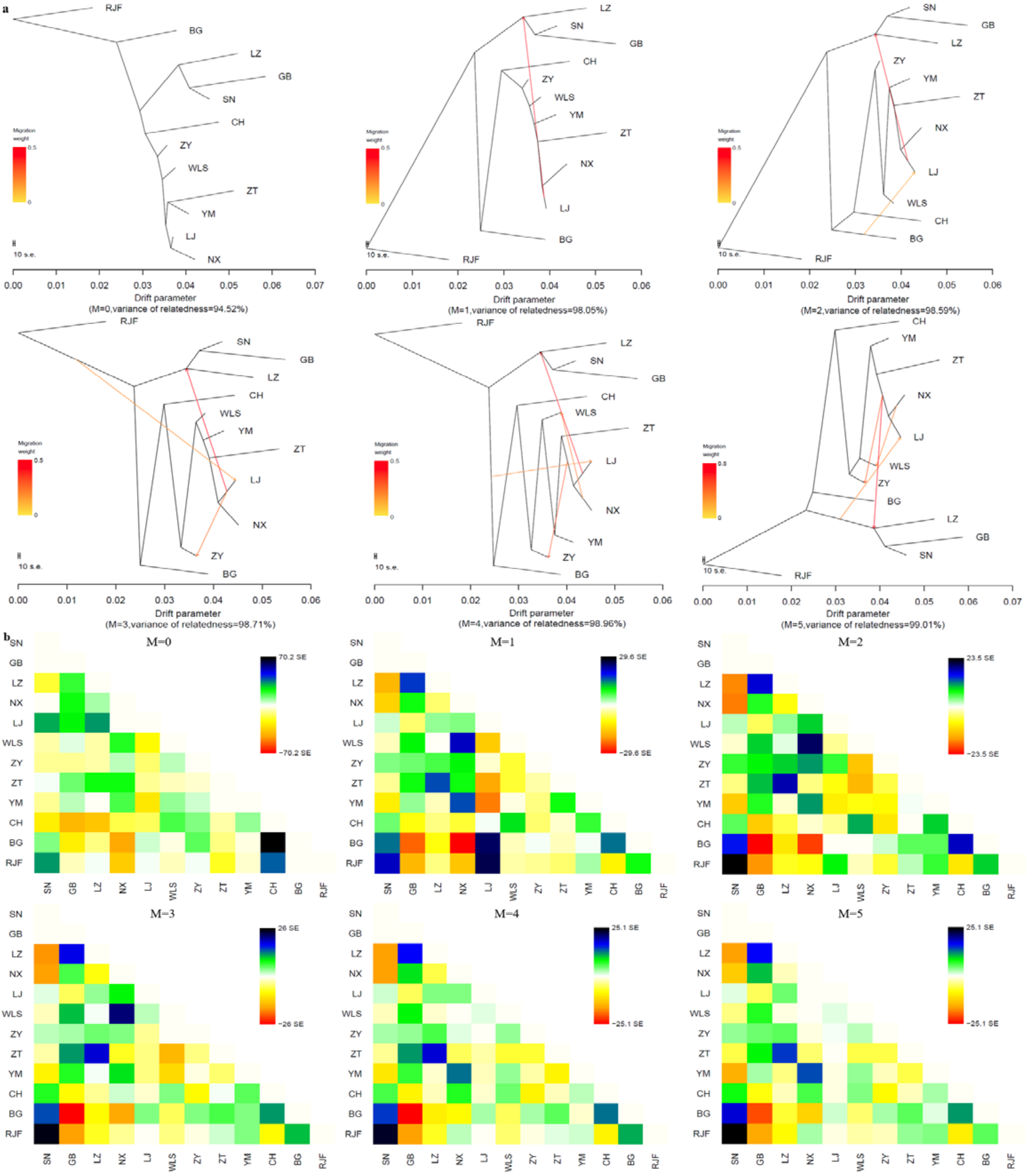

Figure S8. Modeling the history of Tibetan chicken using the qpGraph program. Best fit model inferred by qpGraph program for chicken populations from “1500 m”, “2500 m” and “3200 m+”; the “500 m” population was used as the outgroup. Branch lengths are shown in units of FST × 1000. Dotted lines denote admixture events and values beside the dotted line correspond to admixture proportions.

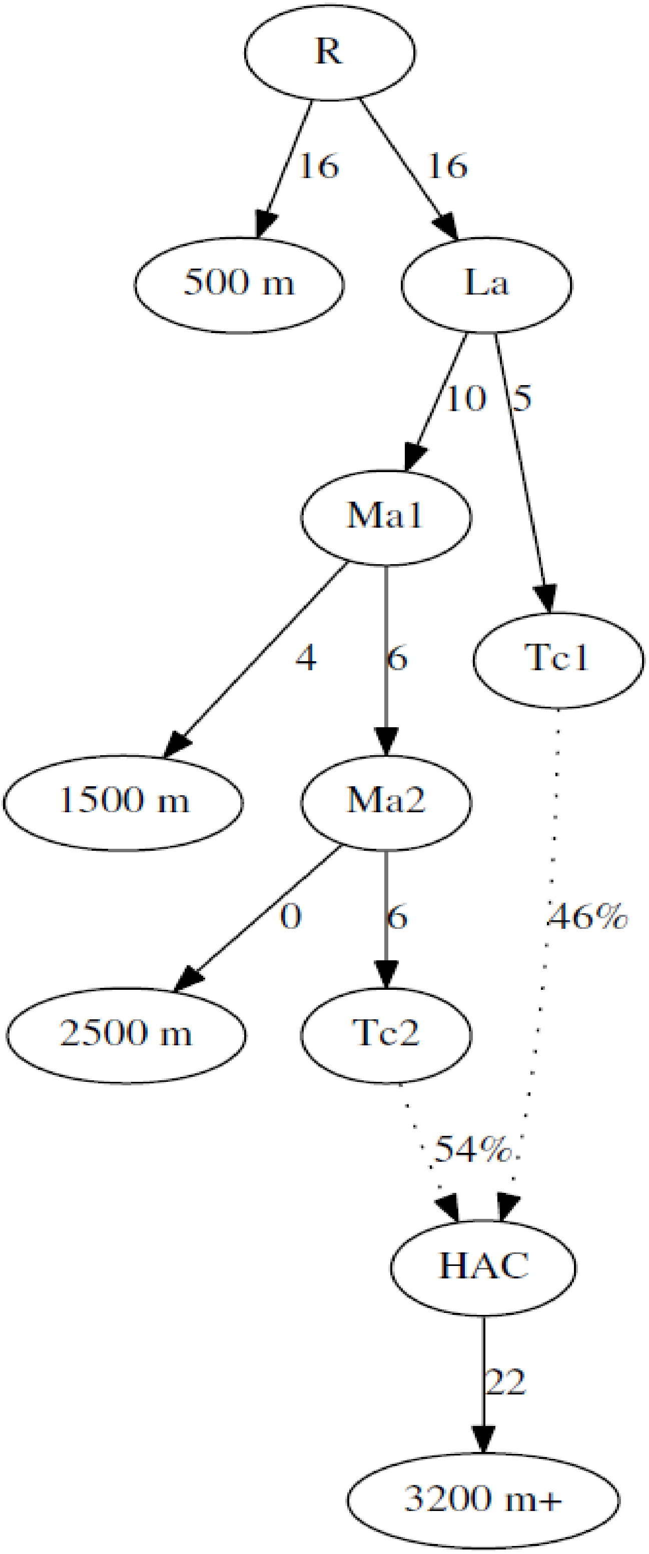

Figure S9. Three-population test between TBC and other Chinese domestic chicken breeds. Estimated *f*3 value ± standard errors are plotted.

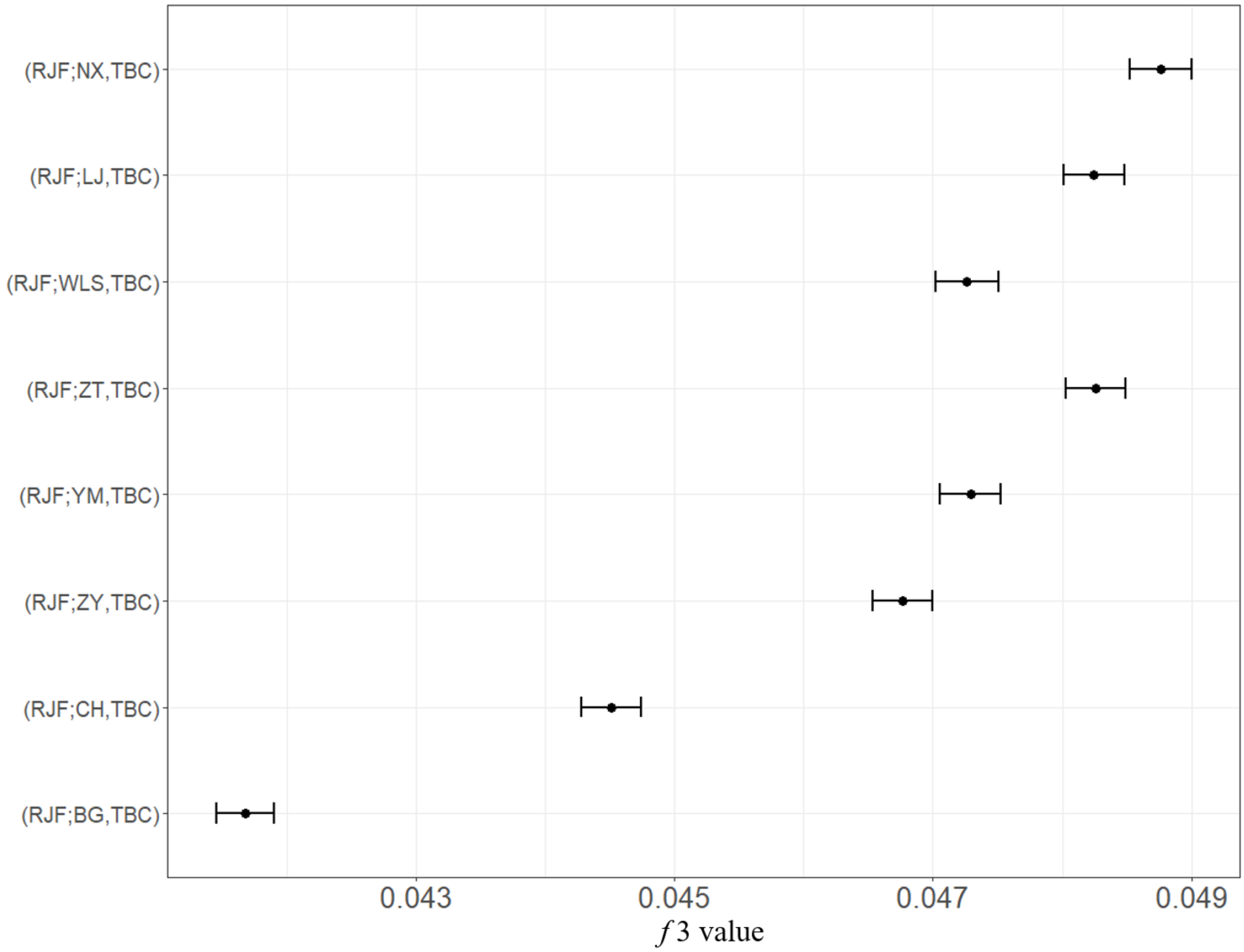

Figure S10. *D* statistics with form D (W, X; Y, Z) for TBC and NX. Estimated *D*-value ± standard errors and *Z*-value are plotted.

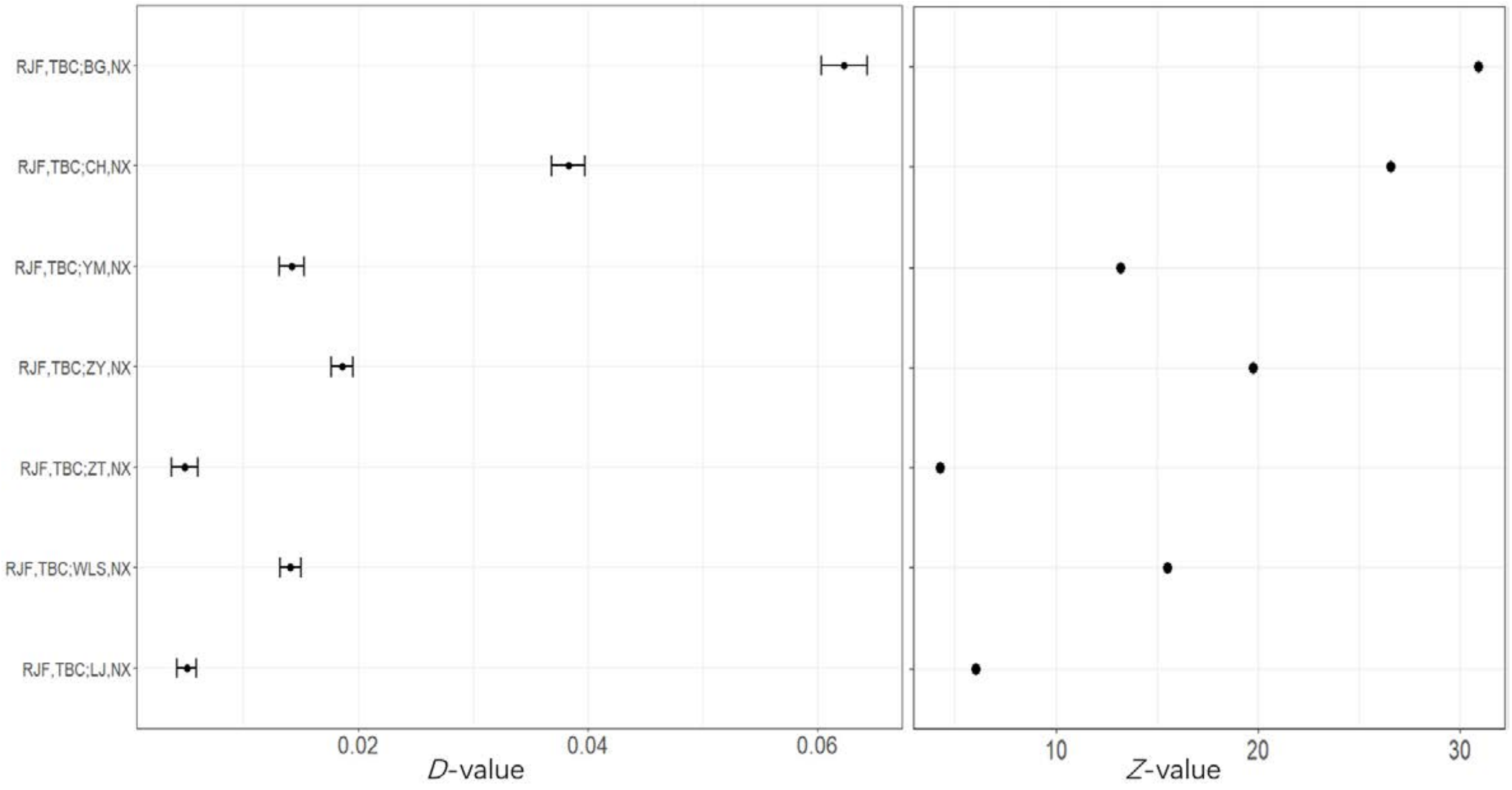

Figure S11. SMC++ estimated effective population size of 12 chicken breeds. Generation time and mutation rate for chicken were 1 year and 1.91 x 10^{-9} substitutions per site per year, respectively.

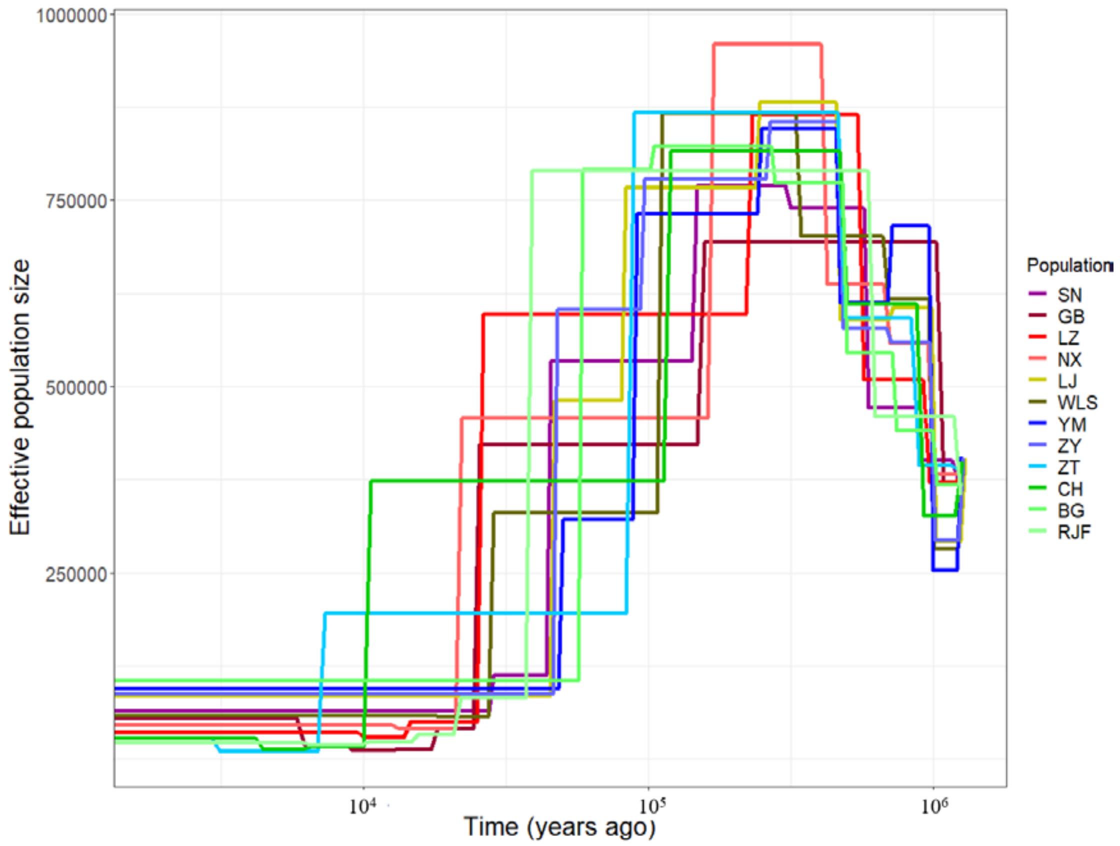

Figure S12. Four alternative models for the demographic history of Tibetan chickens. T0, time when the ancestral Chinese domestic chicken evolved into 1500m altitude area, in years; Tm, time of admixture between populations, in years. In Model 1, Model 2, and Model 3, T1 represents the second split time of ancestral Chinese domestic chicken in years. In Model 1, T2 represents the splitting time of JR and TBC population in years. In Model 2, T2 represents the splitting time of the JR population in years. In Model 3, T2 represents the splitting time of the TBC population in years. In Model 4, T1 represents the splitting time of the JR population while T2 corresponds to the splitting time of the TBC population in years.

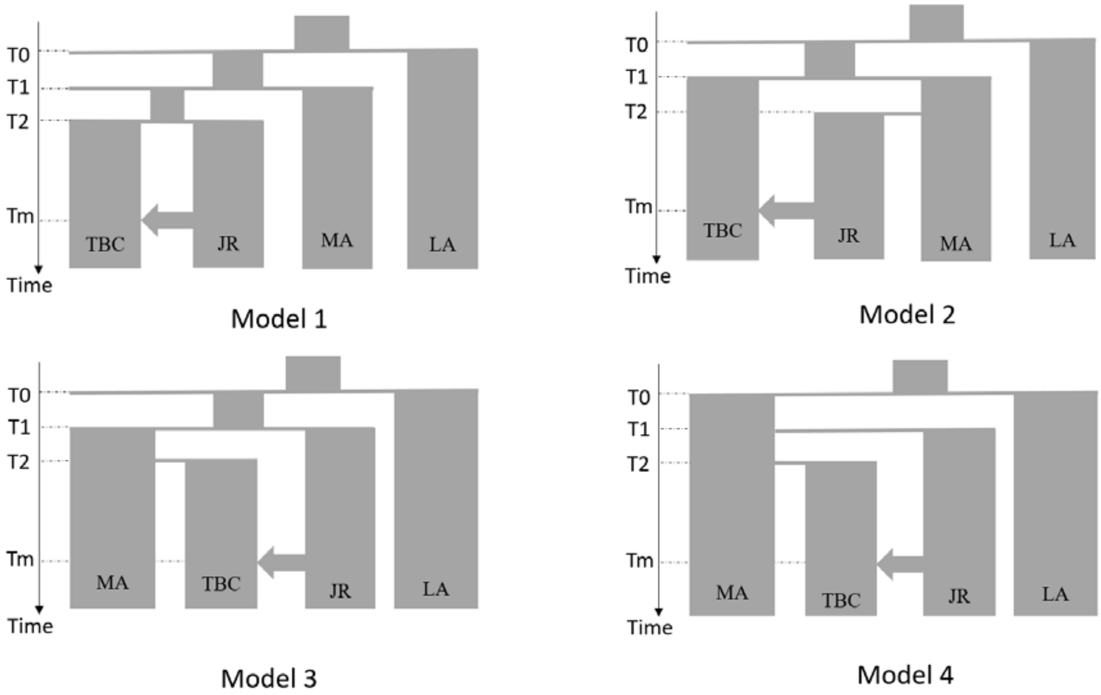

Figure S13. Akaike information criterion (AIC) distribution generated under alternative models. The “****” presents *p*-value < 0.0001. AIC indicated that Model 2 was the best model.

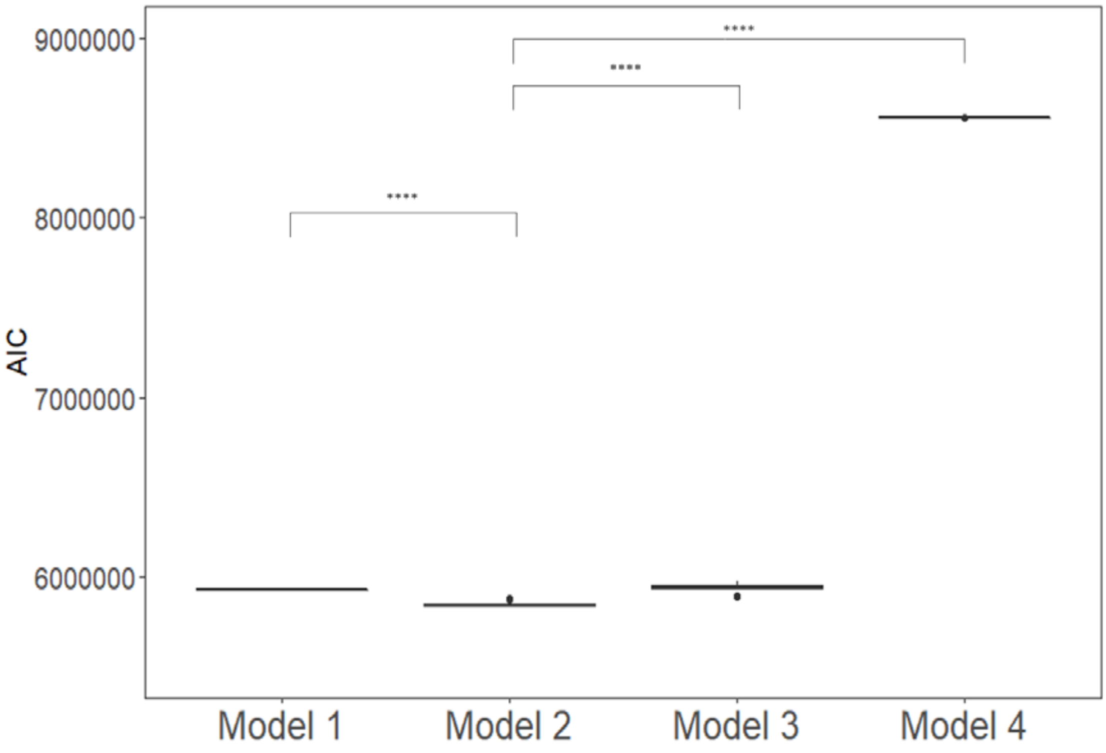

Figure S14. Positive selection scans for high altitude adaptation between TBC and other altitude populations by win-Fst. (a) represents "1500m" and (b) for "2500m". Red line, selection criteria for test value.

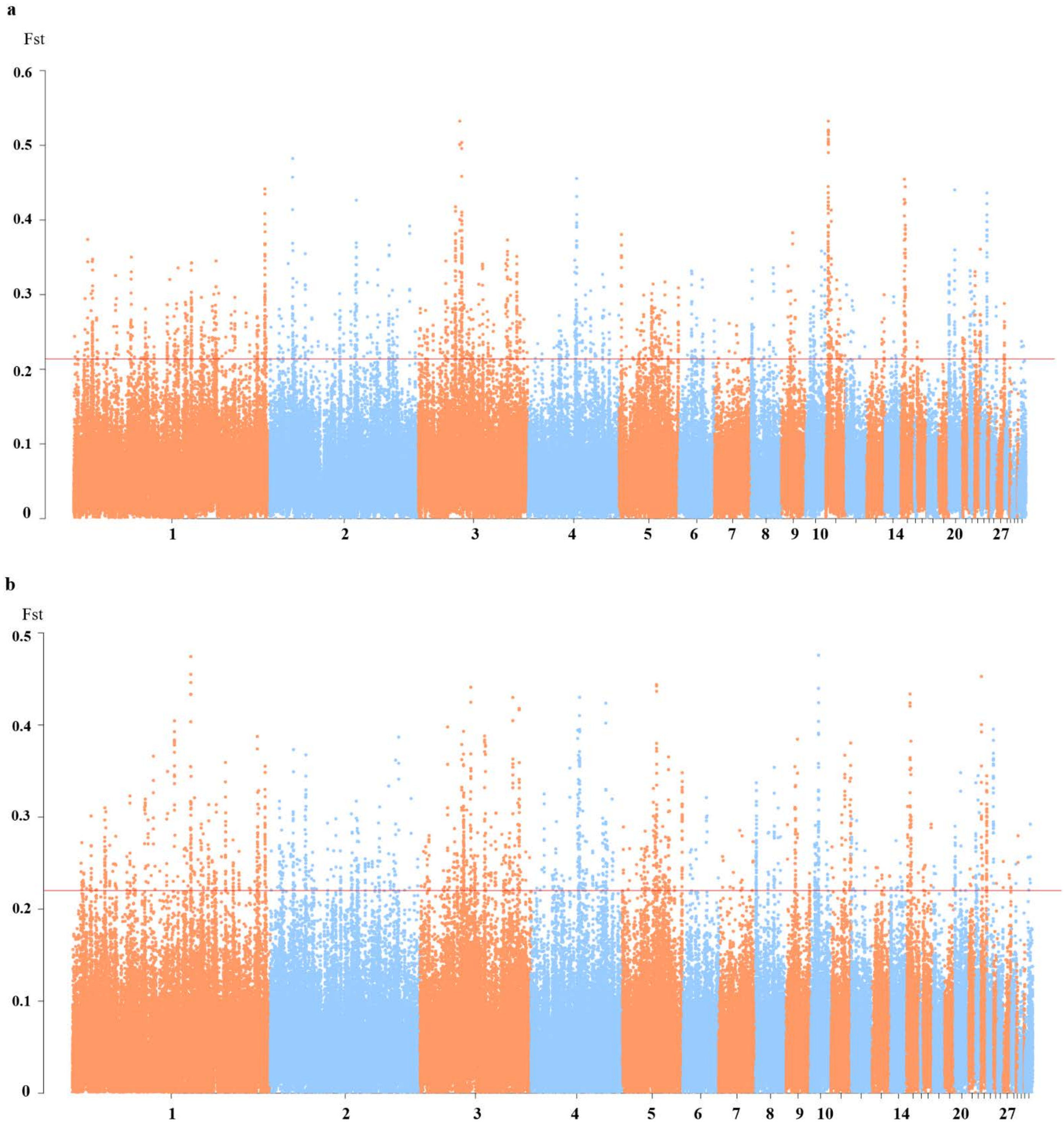

Figure S15. Positive selection scans for high altitude adaptation in NX by win-Fst. Red line, selection criteria for test value; black dots, strongly selected windows.

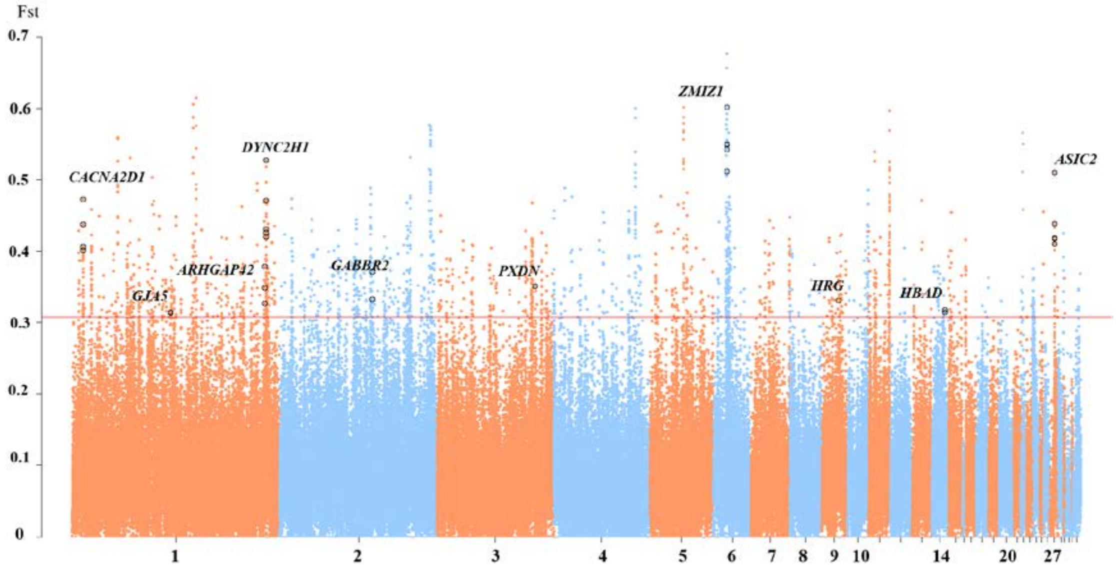

Supplement: Supplementary file 1 — Figure S1 [file EVA-15-2100-s001.pdf]
